# Effects of pristine and citrate-coated zinc oxide nanoparticles on soil nitrogen cycling determined using multi-level assessment of enzyme activity, functional gene abundance and microbial community composition

**DOI:** 10.1007/s11356-026-38042-x

**Published:** 2026-07-15

**Authors:** Ahmed Hussain, Mohammad Jahid Hasan, Kiran Vadde, Akanksha Matta, Matthew Moreno, Esteban E. Ureña-Benavides, Vikram Kapoor

**Affiliations:** 1 School of Civil & Environmental Engineering, and Construction Management, The University of Texas at San Antonio, San Antonio, TX 78249 USA; 2 Department of Biomedical Engineering and Chemical Engineering, The University of Texas at San Antonio, San Antonio, TX 78249 USA; 3 Department of Chemistry, The University of Texas at San Antonio, San Antonio, TX 78249 USA

**Keywords:** Metal oxide nanoparticles, Nitrifying bacteria, Soil, Nitrogen cycle, Ammonia monooxygenase, QPCR

## Abstract

Zinc oxide (ZnO) nanoparticles (NPs) are increasingly detected in agricultural soils, yet their long-term effects on soil nitrogen cycling microorganisms, particularly how surface chemistry modulates toxicity, remain poorly understood. These nanoparticles frequently enter agricultural soils directly using nanopesticides and fertilizers or indirectly through irrigation or sewage sludge application, potentially harming soil microorganisms. This study investigates the effects of pristine and citrate-coated ZnO NPs on the physiological and genetic responses of nitrifying bacteria in soil microcosms. Pristine and citrate-coated ZnO NPs were synthesized in-house and introduced to soil at three concentrations: low (0.01 mg/g), medium (0.1 mg/g), and high (0.5 mg/g). Over a 105-day period, enzyme assays (ammonia monooxygenase [AMO], nitrite oxidoreductase [NXR], nitrite reductase [NIR], hydroxylamine dehydrogenase [HAO]), qPCR of functional genes *(amoA*, *arch-amoA*, *nirK*, *norA*, *hao*), and 16S rRNA gene sequencing were used to track microbial responses. Results showed concentration and citrate-dependent effects. AMO and NXR activities increased early in citrate-coated ZnO NPs and high treatments but declined at later stages, while NIR gradually decreased. Gene abundance patterns supported these trends, with citrate-coated ZnO NPs amplifying *amoA* and *arch-amoA*. Sequencing data indicated substantial shifts in microbial community structure, with notable differences in alpha and beta diversity. Untreated soils maintained higher richness and evenness, whereas high-concentration treatments, particularly citrate-coated ZnO NPs, reduced microbial diversity and altered community clustering. Functional groups responded unevenly, with ammonia- and nitrite-oxidizers increasing while nitrogen-fixers and denitrifiers declined. These findings suggest that ZnO nanoparticles, particularly in citrate-coated form at higher concentrations, may disturb soil microbial functions and shift nitrogen cycling pathways. This highlights potential ecological risks of nanoparticle accumulation in agricultural soils.

**Graphical abstract:**

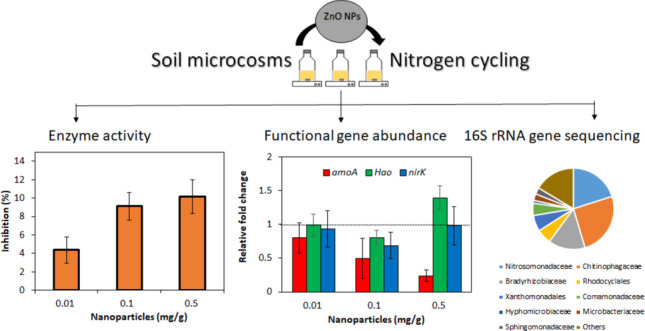

**Supplementary Information:**

The online version contains supplementary material available at 10.1007/s11356-026-38042-x.

## Introduction

The increasing use of metal oxide nanoparticles (MeO NPs) in various industries has raised significant concerns regarding their environmental fate and impact. Among these, zinc oxide (ZnO) nanoparticles are widely utilized due to their applications in agrochemical formulations, protective coatings, electronics, and antimicrobial agents (Beek et al. 2004; Khot et al. 2012). Additionally, they play a crucial role in emerging technologies such as solar cells, sensors, and photocatalytic water treatment processes (Majumder et al. 2020; Ullah and Dutta 2008). However, their widespread application has led to their unintended release into the environment through multiple pathways, including wastewater discharge, sewage sludge amendments, and direct soil application as nanofertilizers and nanopesticides (DeRosa et al. 2010; Dubey and Mailapalli 2016; Kapoor et al. 2018; Lombi et al. 2012; Yang et al. 2014). Recent studies suggest that concentrations of ZnO NPs in soils have been increasing (Rajput, et al. 2018; Solymos et al. 2025; Wu 2019), and this uncontrolled environmental release raises substantial concerns about their potential effects on soil microbial communities, particularly those involved in essential biogeochemical processes such as the nitrogen (N) cycle.

Soil nitrogen cycling is largely governed by microbial processes, particularly nitrification and denitrification, which regulate soil fertility and plant nutrient availability (Ben-Moshe et al. 2013; Frenk et al. 2013). Nitrification involves the oxidation of ammonia to nitrite by ammonia-oxidizing bacteria (AOB) and its subsequent oxidation to nitrate by nitrite-oxidizing bacteria (NOB). While some *Nitrospira* species have been found to perform complete ammonia oxidation (Daims et al. 2015), nitrification is typically rate-limited by the slow growth and environmental sensitivity of AOB and NOB (Kapoor et al. 2015; Li et al. 2016; Prosser 1990; Wang et al. 2015). These microbes are particularly vulnerable to contaminants, including ZnO NPs (Kapoor et al. 2018; Phan et al. 2020), which may inhibit their activity and disrupt nitrogen availability in soils. Despite growing concerns, the mechanisms underlying ZnO NP uptake and toxicity in nitrifying bacteria remain poorly understood. Most research has focused on pure cultures of AOB, such as *Nitrosomonas europaea*, which have demonstrated nanoparticle-induced inhibition of ammonia oxidation (Radniecki et al. 2011; Yu et al. 2015). However, these studies do not account for the complex microbial interactions that occur in natural soils, where diverse bacterial communities may exhibit varying degrees of resilience or susceptibility to nanoparticle exposure. A more comprehensive understanding of how ZnO NPs influence soil microbial dynamics is crucial for predicting ecosystem responses and informing sustainable nanoparticle applications.

In the soil environment, few studies have focused on the impact of ZnO NPs on microbial structure and function. High concentrations of ZnO NPs (1000 mg/kg) increased the functional gene abundance of AOB and nitrite reductase (*nirS*), while reducing ammonia-oxidizing archaea (Liu et al. 2021). A recent review further confirmed that ZnO NPs consistently reduce the abundance of microbial communities responsible for ammonia oxidation and nitrite reduction across diverse soil types, while stimulating certain bacterial genera at low concentrations (Strekalovskaya et al. 2024). These gene-level shifts suggest enhanced nitrification and denitrification activity, linking ZnO NPs to higher N_2_O release. Notably, ammonia-oxidizing archaea have been shown to exhibit greater resilience to metal stress than their bacterial counterparts, owing to differences in cell envelope composition and ecological strategy (Prosser and Nicol 2012), suggesting that bacterial and archaeal nitrifiers may respond differentially to ZnO NP exposure. Other studies report broader microbial and enzymatic disruptions due to the presence of nanoparticles. ZnO and Fe_2_O_3_ NPs reduced bacterial and fungal populations, suppressed nitrogen mineralization, and lowered radish yield. Biogenic ZnO NPs also temporarily reduced microbial biomass but increased soil respiration, while long-term exposures shifted communities of *Nitrospirae* and *Actinobacteria*, phyla central to nitrogen transformations (Chen et al. 2021; Mahmood et al. 2021). Together, these findings show ZnO NPs disrupt enzyme-driven processes in nitrogen cycling, raising concerns for both soil fertility and greenhouse gas emissions. However, the differential sensitivity of distinct nitrogen-cycling functional groups, including nitrifiers and denitrifiers, to ZnO NP stress remains poorly characterized, particularly under long-term chronic exposure conditions. Despite these findings, most existing studies on the impact of ZnO NPs on soil microbial communities remain limited (Shirvani and Ghalandari 2024; Shrivastava et al. 2019). Many lasted only a few weeks (≤ 30 days) or focused solely on community composition without connecting gene abundance to enzyme activity. Very few studies have investigated both molecular and biochemical responses together over a long period, and fewer still have compared the effects of pristine versus surface-modified ZnO NPs on nitrogen cycling functional guilds simultaneously. Additionally, the influence of citrate coating, one of the most common surface modifications applied to engineered nanoparticles, on soil nitrogen cycling microorganisms remains poorly characterized. This leaves important gaps in understanding the resilience of soil microbes under chronic nanoparticle stress, whether surface chemistry modulates toxicity pathways differently across functional guilds, and whether nitrogen cycling can fully recover after exposure. Furthermore, recent work highlights that soil physicochemical properties such as texture significantly mediate ZnO NP toxicity to soil microbial activity (Shah et al. 2025), underscoring the importance of soil-specific assessments (Shah et al. 2025).

This study investigates the effects of ZnO NPs on soil nitrifying bacteria through a controlled microcosm experiment. Three treatment groups were established with varying concentrations of ZnO NPs (low 0.01 mg/g, medium 0.1 mg/g, and high 0.5 mg/g of soil), including both pristine and citrate-coated nanoparticles to assess the influence of surface chemistry on microbial interactions. Based on the evidence reviewed above, we propose three hypotheses. First, ZnO NP exposure will inhibit nitrification enzyme activities (AMO, HAO, NXR) and denitrification enzyme activity (NIR) in a concentration-dependent manner, with citrate-coated NPs producing greater effects than pristine NPs possibly due to enhanced Zn^2^⁺ bioavailability (Radniecki et al. 2011; Yu et al. 2015). Second, bacterial *amoA* abundance will be more adversely affected than *arch-amoA*, reflecting greater stress tolerance in ammonia-oxidizing archaea (Prosser and Nicol 2012), and gene abundance changes will not fully mirror enzyme activity responses due to post-transcriptional regulation and functional redundancy. Third, ZnO NP exposure will restructure microbial community composition in a concentration- and citrate-dependent manner, with nitrogen-fixing and denitrifying functional groups disproportionately affected relative to nitrifying taxa, reflecting differential sensitivity across nitrogen-cycling functional guilds rather than a uniform decline in community diversity. Soil samples were incubated over 105 days and assessed at five time points (21-day intervals) to evaluate both short- and long-term effects on microbial structure and function. Enzymatic assays were performed to assess the functional activity of nitrifying bacteria, while quantitative polymerase chain reaction (qPCR) assays and 16S rRNA gene sequencing provided insights into functional gene abundance and shifts in microbial community composition. By integrating molecular and biochemical approaches, this study aims to elucidate the impacts of ZnO NP exposure on soil nitrification and microbial resilience, contributing to a broader understanding of nanoparticle toxicity and its environmental implications.

## Materials and methods

### Soil collection

Soil was collected from the upper 15 cm of an agricultural field in San Antonio, Texas (29.4241° N, 98.4936° W), a semi-arid region with well-drained soil. The collected soil was classified as sandy loam (55% sand, 20% silt, 25% clay), and a pH of 7.9, electrical conductivity of 222.1 µS/cm, total organic carbon (TOC) of 37.7 mg/kg dry soil, nitrate-N of 7.65 mg/kg dry soil, nitrite-N of 0.48 mg/kg dry soil, and ammonia-N of 0.09 mg/kg dry soil. The soil was air-dried at room temperature (25 °C) and sieved through a 2-mm mesh to remove debris and used immediately.

### Nanoparticle preparation and characterization

Pristine ZnO NPs were synthesized in-house by hydrolysis of zinc acetylacetonate in 1,4-butanediol, following Visinescu et al. (). Citrate-coated ZnO NPs were prepared by adding sodium citrate during the reaction at defined citrate/ZnO molar ratios. Citrate was selected as the coating agent because it is a naturally occurring organic acid commonly found in soils and plant root exudates, and because citrate coating has been shown to alter nanoparticle surface charge, colloidal stability, and dissolution kinetics, potentially modulating Zn^2+^ bioavailability and toxicity to soil microorganisms (Akhil and Sudheer Khan 2017; Silina et al. 2024). Details of the synthesis are provided in Supplementary Information**.** Nanoparticle morphology and structure were characterized using transmission electron microscopy (TEM), X-ray diffraction (XRD), and Raman spectroscopy. Surface charge and stability were determined by zeta potential and dynamic light scattering (DLS). Details regarding TEM, XRD, DLS, and Raman spectroscopy are provided in Supplementary Information**.** Fourier-transform infrared (FTIR) spectroscopy was performed on the pristine ZnO and citrate-coated ZnO NP samples using a Shimadzu IRAffinity-1S spectrometer (Shimadzu Corporation, Kyoto, Japan). To enhance the signal-to-noise ratio, each spectrum was obtained by averaging 100 scans.

### Experimental design and soil treatment

Soil microcosms were set up in 250 mL Erlenmeyer flasks, each containing 100 g of soil and 40 mL of Milli-Q EQ 7000 Ultrapure water, bringing the soil moisture to its water-holding capacity. The experimental design comprised seven distinct treatment conditions: a single control treatment with only pure Milli-Q water, and six nanoparticle treatments—three with ZnO nanoparticles (0.01, 0.1, and 0.5 mg/g of soil) and three with citrate-coated ZnO nanoparticles at the same concentrations. The selection of exposure concentrations was based on several considerations. The low concentration (0.01 mg/g) represents environmentally relevant levels that may accumulate in agricultural soils through repeated applications of biosolids, nanofertilizers, or nanopesticides (García-Gómez et al. 2020; Ge et al. 2011). The medium concentration (0.1 mg/g) reflects projected accumulation scenarios over extended periods of nanoparticle-amended biosolid applications to agricultural land and has been widely used in previous soil microbial studies to assess sub-lethal effects (Chen et al. 2021; Frenk et al. 2013; Liu et al. 2021). The high concentration (0.5 mg/g) represents a worst-case scenario for chronic nanoparticle accumulation and was selected to identify threshold toxicity effects on nitrogen-cycling microorganisms (Chen et al. 2021; Liu et al. 2021; Shah et al. 2022). Together, these concentrations span a range from realistic environmental projections to stress-inducing levels, allowing us to capture both subtle and pronounced concentration-dependent effects on soil nitrogen cycling.

Nanoparticles were first dispersed in 5 mL vials with deionized water and ultrasonicated to break up aggregates and ensure a stable suspension. The prepared suspensions were then introduced dropwise to the soil while the flasks were continuously mixed on a vortex mixer. This systematic approach facilitated comparative analysis of dose-dependent responses and the influence of surface functionalization on nanoparticle bioavailability and subsequent toxicological effects within the soil matrix. The flasks were sealed with funnels and incubated in the dark at 25 ± 2 °C to keep the moisture levels steady (40–50% of water-holding capacity). The microcosms were opened weekly for 5 min for aeration before being sealed again. Each treatment was performed in triplicate, resulting in 21 microcosms per time point across five time points, for a total of 105 microcosms. The experiment ran for 105 days, with samples collected every 21 days. The 21-day sampling interval was selected to capture both short-term stress responses and longer-term adaptive or recovery dynamics in the microbial community, while remaining practical for destructive sampling across 105 microcosms. The 105-day total duration was chosen to extend beyond most previously reported ZnO NP soil studies (typically 30 days or less), enabling detection of delayed or chronic effects on nitrogen cycling microorganisms.

### Soil sampling and storage

Soil samples were collected at five time points (days 21, 42, 63, 84, and 105). At each time point, a destructive sampling method was used, meaning each microcosm was fully used to collect samples: 0.25 g of soil for each DNA extraction in 2 ml nuclease free tube and 1 g of soil for each enzymatic assay in 15 ml polypropylene centrifuge tubes. Samples designated for enzyme assays were stored at −80 °C, while samples for DNA extractions were stored at −20 °C.

### Enzyme activity assays

To determine the effect of ZnO NPs on nitrogen transformation at the protein level, key enzyme activities involved in nitrogen cycling were measured at designated intervals. These included ammonia monooxygenase (AMO), hydroxylamine dehydrogenase (HAO), nitrite oxidoreductase (NXR), and nitrite reductase (NIR). The activities of AMO, NXR, and NIR were quantified using spectrophotometric methods with minor modifications based on enzyme activity in this study (Chen et al. 2019, 2023; Ma et al. 2021). AMO activity was assessed by measuring the oxidation of ammonium to nitrite, while NXR activity was evaluated by measuring the oxidation of nitrite to nitrate in phosphate-buffered solutions containing specific substrates. NIR activity was determined under anaerobic conditions by measuring the reduction of nitrate to nitrite and nitrite to nitric oxide, respectively. HAO activity was assessed by monitoring the oxidation of hydroxylamine. All enzyme activity assays were performed in triplicate, maintaining consistent incubation conditions (37 °C, 200 r/min) to ensure reproducibility. Enzyme activity for each treatment was expressed as relative activity (%), calculated as follows: Relative Activity (%) = (*A*_treatment, t_/*A*_control, t_) × 100, where *A*_treatment, t_ is the mean absorbance of the NP-treated microcosm and *A*_control, t_ is the mean absorbance of the untreated control (0 mg/kg soil) measured at the same time point *t*. Normalization was performed independently for each time point. The detailed procedural steps for enzyme activity assays are provided in Supplementary Information.

### DNA extraction and qPCR assays

Soil microbial DNA was extracted using the DNeasy PowerSoil PowerLyzer Kit (Qiagen) following the manufacturer’s instructions, with 250 mg of soil used per sample. DNA purity and concentration were measured using a NanoDrop One^C^ spectrophotometer (Thermo Fisher Scientific).

The occurrence and relative abundance of five qPCR markers were used to assess the abundance of key functional genes involved in nitrification and denitrification (Table S1). The genetic markers included primer sets targeting archaeal *amoA*, bacterial *amoA*, *nirK*, *norA*, and *hao* genes (Francis et al. 2005; Henry et al. 2004; Leung et al. 2022; Rotthauwe et al. 1997). All qPCR reactions were carried out in triplicate on the CFX96 Touch Real-Time PCR Detection System (Bio-Rad, Hercules, CA) using SsoAdvanced Universal SYBR Green Supermix (Bio-Rad). No template controls were used to check for cross-contamination. Primer specificity and the absence of primer-dimers were confirmed via melting curve analysis for each qPCR assay conducted. Additionally, absence of PCR inhibition was tested and confirmed in DNA extracts by comparing *C*_T_ values of undiluted and 10-fold dilutions of DNA extracts as qPCR template. Functional gene abundances were quantified using the ΔΔ*C*_t_ relative quantification method (fold change = 2^(− ΔΔCt)^), normalizing target gene *C*_t_ values to the reference gene (16S rRNA gene) (Parada et al. 2016) within each sample and to the mean Δ*C*_t_ of the untreated control at each time point.

### 16S rRNA gene sequencing

The genomic DNA extracted from untreated and treated soil samples was analyzed for bacterial, archaeal, and eukaryotic composition through next-generation sequencing using the AVITI platform from Element Bioscience (San Diego, CA). The improved primer set targeting V4 and V5 hypervariable regions of the bacterial 16S rRNA gene, which is capable of amplifying about 400 bp fragments of bacterial and archaeal 16S rRNA and around 600 bp fragments of fungal 18S rRNA, was utilized for sequencing (Table S1) (Milke et al. 2022; Parada et al. 2016). PCR amplification and sequencing were conducted at the Genomics Core Facility at the University of Texas at San Antonio (UTSA), San Antonio, TX, USA. PCR reactions were carried out in triplicate for each treatment with the barcoded primer set (515F and 926R) in a 25 µL reaction volume, which included 2 × NEBNext® Ultra™ II Q5® Master Mix (New England Biolabs, Ipswich, MA), 1 µM of both forward and reverse primers, and 5 ng of template DNA. The cycling conditions for the PCR consisted of an initial denaturation at 95 °C for 3 min, followed by 25 cycles of 95 °C for 45 s, 50 °C for 45 s, and 68 °C for 90 s, with a final extension at 68 °C for 5 min. The PCR products were analyzed on a 2% agarose gel, purified using the AMPure XP beads kit (Beckman Coulter, USA), and quantified using the Qubit dsDNA HS Assay Kit (Thermo Fisher Scientific, USA) according to the manufacturer’s instructions. The purified PCR products from each treatment group were combined in equimolar concentrations to create gene libraries with the Elevate Enzymatic Library Prep Kit (Element Bioscience, San Diego, CA), followed by sequencing using the AVITI benchtop system with paired-end 300 bp Cloudbreak sequencing kits from Element Bioscience. In total, 36 samples (comprising 6 positive controls collected at 0, 21, 42, 63, 84, and 105 days and 15 samples each from ZnO and citrate-coated ZnO NPs treated soils at low, medium, and high concentrations collected at 21, 42, 63, 84, and 105 days) were sequenced and analyzed for microbial community composition using NGS.

The generated sequences were analyzed using the bioinformatics pipeline developed by Quantitative Insights into Microbial Ecology 2 (QIIME2) (Bolyen et al. 2019). Forward and reverse primers were discarded using cutadapt, and DADA2 was utilized for the trimming and truncation of paired-end sequences, as well as for denoising, merging reads, and removing chimeras (Callahan et al. 2016). Representative sequences from DADA2 were used to create a phylogenetic tree with Mafft (Katoh), which enabled the calculation of diversity metrics using the core-metrics-phylogenetic plugin of QIIME2. This plugin also generated a weighted Unique Fraction (UniFrac) distance matrix that incorporates phylogenetic information based on the presence and relative abundance of amplicon sequence variants (ASVs) that were generated through DADA2 (Lozupone et al. 2011), and the data were subsequently exported from QIIME2 for further statistical analysis. To enhance the quality of taxonomic assignment, a feature classifier was trained using reference sequences from the SILVA database (version 132) with the primer set used for sequencing. This classifier was then employed by VSEARCH to determine the taxonomy of ASVs with 99% similarity. The raw sequences generated in this study were deposited in the National Center for Biotechnology Information (NCBI) Short Read Archive (SRA) database under the accession numbers PRJNA146277.

### Statistical analyses

The results for enzyme activity assays were expressed as relative percent activity, and the results for qPCR were represented as fold change in relative gene abundance. All experiments were performed as experimental triplicates. The normality and homogeneity of variance of the enzyme activity and functional gene fold-change datasets were evaluated using the Shapiro-Wilk and Levene’s tests, respectively. Results indicated that the data did not follow a normal distribution (Shapiro-Wilk, *p* < 0.05, Table S2) and exhibited unequal variance among treatment groups (Levene’s test, *p* < 0.05). Due to these violations of parametric assumptions, non-parametric statistical approaches were employed: the Kruskal-Wallis test was used to assess overall differences among treatment groups at each time point, followed by Dunn’s test for pairwise post-hoc comparison between each treatment and the untreated control, with Benjamini-Hochberg false discovery rate (FDR) correction applied to account for multiple comparisons. Alpha diversity, indicative of species richness and evenness within samples, was measured through Faith's phylogenetic diversity (PD), Pielou’s evenness, and Shannon (H′) diversity indices (Chao et al. 2016). The weighted UniFrac distance matrix, generated through QIIME2 core metrics plugin, was imported into the R environment to analyse beta diversity through hierarchical cluster analysis (HCA) and principal coordinate analysis (PCoA) for identifying the differences and relationships in microbial communities among untreated and treated soil samples (Moghadam et al. 2022). The statistical significance of the alpha bacterial diversity indices was assessed using the Kruskal-Wallis non-parametric test (Pérez-Valdespino et al. 2021). Permutational multivariate analysis of variance (PERMANOVA) using the adonis2 function in the vegan package in R was performed on PCoA data, with 999 permutations to evaluate the effects of treatment type, concentration, and day on microbial community structure. Each factor's contribution to variance was expressed as *R*^2^ values, with statistical significance established at *p* < 0.05 (Bao et al. 2022). The ALDEx2 package in R was used to analyze differential abundance and identify bacterial genera significantly affected by ZnO and citrate-coated ZnO NP treatments relative to the positive control. ALDEx2 used a centered log-ratio (CLR) transformation, along with Monte Carlo sampling, to model compositional aspects of amplicon sequencing data. Statistical significance was assessed through Welch’s *t*-test with Benjamini–Hochberg FDR correction, identifying taxa with adjusted *p*-values below 0.05 as significant (Fernandes et al. 2013; Nearing et al. 2022).

## Results and discussion

### Characterizations of ZnO nanoparticles

**Fig. 1:**
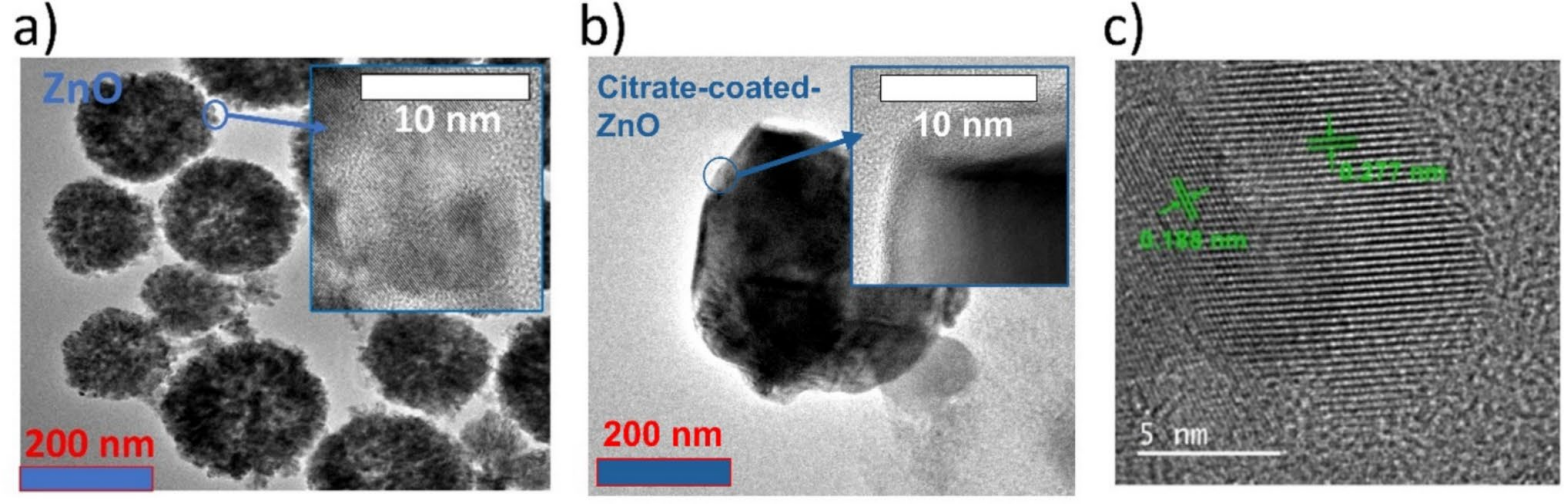
TEM images of (**a**) pristine ZnO NPs; (**b**) citrate-coated ZnO NPs; (**c**) high magnification image of pristine ZnO NPs

Transmission electron microscopy (TEM) images (Fig. 1) revealed that the ZnO NPs (Fig. 1a) are spheroidal, with individual sizes ranging between 10 and 15 nm. However, they tend to form larger clusters measuring approximately 100 to 200 nm. These findings are in line with the results reported by Visinescu et al. (), who observed 12 nm crystallites forming aggregates of 50 to 100 nm using the polyol hydrolysis method. The citrate-coated ZnO NPs (Fig. 1b) were found to range from 100 to 300 nm, with the aggregates appearing more densely packed. Figure 1c provides a high-resolution image of the ZnO NPs, clearly displaying the crystalline lattice fringes and interlayer spacing, which aligns with the atomic distances associated with a ZnO wurtzite-type phase (Visinescu et al. 2015). While these observations suggest a wurtzite structure, further X-ray diffraction analysis is required to confirm the crystalline phase and assess the purity of the materials.

**Fig. 2:**
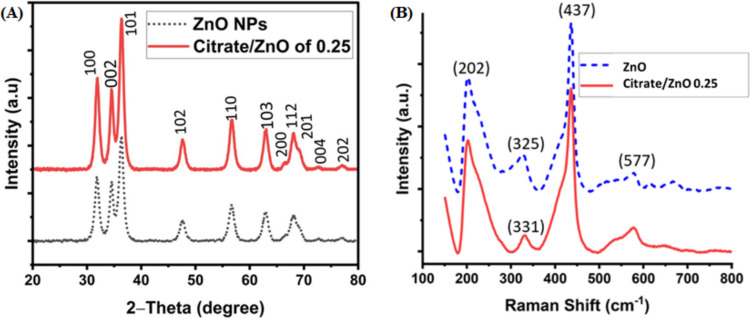
**(A)** XRD of ZnO and citrate-coated ZnO NPs; (**B**) Raman spectroscopy ZnO and citrate-coated ZnO NPs. The '0.25' refers to the citrate-to-ZnO molar ratio used in the synthesis of citrate-coated ZnO NPs

The X-ray diffraction (XRD) patterns of ZnO and citrate-coated ZnO 0.25 NPs are shown in Fig. 2A. The ZnO NPs exhibited distinct diffraction peaks corresponding to the crystallographic planes (100), (002), (101), (102), (110), (103), (200), (112), (201), (004), and (202), characteristic of the ZnO wurtzite-type phase, consistent with the findings of Visinescu et al. (Visinescu et al. 2015). The XRD diffractogram of the citrate-coated ZnO 0.25 NPs displayed identical peaks, indicating that the citrate coating did not alter the crystalline structure of the ZnO NPs. Raman spectroscopy was employed to further characterize the structure of ZnO and citrate-coated ZnO NPs. The spectra, obtained at room temperature using a 532 nm excitation wavelength, are presented in Fig. 2B. Characteristic Raman peaks were observed at 202, 325, 437, and 577 cm^−1^, corresponding to the vibrational modes of the ZnO wurtzite structure, consistent with the published research articles (Abdelouhab et al. 2020; Silambarasan et al. 2015). The prominent peak at 437 cm^−1^ is attributed to the E_2_ (high) mode, a hallmark of the hexagonal wurtzite phase, confirming the crystalline nature of ZnO. The peaks at 202 cm^−1^ and 325 cm^−1^ correspond to the E_2_ (low) mode and A_1_(TO) mode, respectively, while the 577 cm^−1^ peak is associated with the A_1_(LO) mode, typically linked to oxygen vacancies or defects within the ZnO lattice. Notably, the citrate-coated ZnO NPs exhibited identical Raman spectra to the uncoated ZnO, further confirming that the citrate coating did not affect the crystalline structure of the nanoparticles.

To confirm successful surface functionalization, FTIR spectroscopy was performed on both pristine and citrate-coated ZnO NPs (Figure S1a). The FTIR spectrum of pristine ZnO NPs exhibited a characteristic absorption band at ~ 418 cm⁻^1^, which is attributed to the Zn–O stretching vibration, confirming the formation of ZnO (Acharya et al. 2024). The peaks observed in the 400–600 cm⁻^1^ region are typically attributed to metal–oxygen interactions (Gomathi and Suhana 2021; Hasan et al. 2026a, b). The absence of additional significant peaks in the higher wavenumber region indicates minimal organic residues and suggests good purity of the synthesized nanoparticles. In contrast, the citrate-coated ZnO NPs retained the Zn–O band at ~ 418 cm⁻^1^, while showing additional prominent peaks at 1573 and 1394 cm⁻^1^, corresponding to the asymmetric and symmetric stretching vibrations of carboxylate (COO⁻) groups, respectively, confirming the successful citrating coating on ZnO (Doan et al. 2025; Vassallo et al. 2023). Furthermore, the appearance of a broad band around ~ 3400 cm⁻^1^ is assigned to O–H stretching vibrations arising from surface hydroxyl groups, adsorbed water, and hydroxyl functionalities of citrate. These observations collectively confirm the successful surface functionalization of ZnO NPs with citrate.

To investigate the colloidal stability of ZnO NPs, both zeta potential and hydrodynamic diameter were measured (Figure S1b). At pH 7, ZnO NPs exhibited a positive surface charge (⁓ + 28 mV), which remained stable up to pH 9, followed by decreasing to ⁓ −33 mV at pH 11, consistent with the findings reported by Zhang et al. () In contrast, the citrate/ZnO 0.25 NPs displayed zeta potentials of ⁓ + 5, −6, −22, and −32 mV at pH 7.6, 7.9, 9.8, and 11, respectively. Additionally, citrate/ZnO 0.8 NPs showed a surface charge of ⁓ −7 mV at pH 7, decreasing to ⁓ −22 mV at pH 10. These results indicate that citrate coating significantly reduces the surface charge of the NPs, compared to uncoated ZnO NPs. A zeta potential exceeding + 30 mV or below −30 mV typically suggests good colloidal stability. The colloidal stability was further examined by assessing the hydrodynamic diameter of the NPs over time at pH 7 using dynamic light scattering. The initial size of ZnO and citrate/ZnO 0.25 NPs was 346 ± 28 nm and 620 ± 102 nm, respectively. ZnO NPs aggregated significantly over 6 h, reaching sizes > 2000 nm. In contrast, citrate/ZnO 0.25 NPs displayed better stability, with sizes remaining < 1000 nm after 6 h. On the other hand, citrate/ZnO 0.8 NPs showed larger aggregates of approximately 3000 nm at time 0, which slightly increased to 3400 nm after 6 h. Based on these results, ZnO and citrate/ZnO 0.25 NPs were selected for further soil studies due to their better colloidal stability at pH 7 compared to other tested samples.

### Impact of ZnO NPs on enzyme activities

**Fig. 3:**
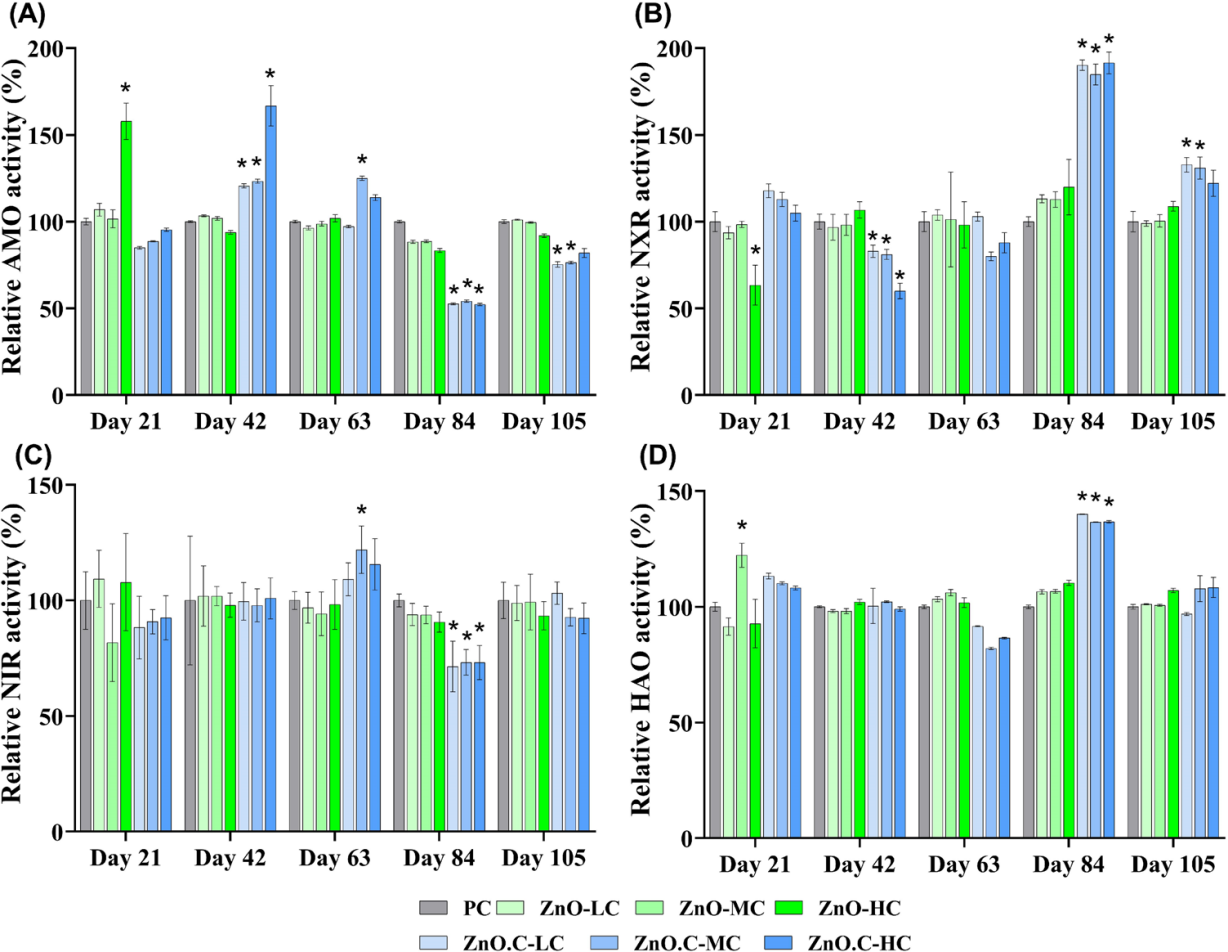
Relative enzyme activity of (**A**) AMO, (**B**) NXR, (**C**) NIR, and (**D**) HAO over time in various treatment groups. ‘PC” stands for positive control, ‘ZnO-LC’ for low concentration of ZnO NPs (0.01 mg/g of soil), ‘ZnO-MC’ for medium concentration of ZnO NPs (0.1 mg/g of soil), ‘ZnO-HC’ for high concentration of ZnO NPs (0.5 mg/g of soil), ‘ZnO.C-LC’ for low concentration of citrate-coated ZnO NPs (0.01 mg/g of soil), ‘ZnO.C-MC’ for medium concentration of citrate-coated ZnO NPs (0.1 mg/g of soil), ‘ZnO.C-HC’ for high concentration of citrate-coated ZnO NPs (0.5 mg/g of soil). Asterisks indicate statistically significant differences from the untreated control at the same time point (Kruskal–Wallis test followed by Dunn's post-hoc test, Benjamini–Hochberg FDR-corrected; *p < 0.05). Data represent mean ± SD of three independent biological replicates (*n* = 3)

The activities of key enzymes involved in ammonification, nitrification and denitrification were measured throughout the entire experiment, including ammonia monooxygenase (AMO), hydroxylamine dehydrogenase (HAO), nitrite oxidoreductase (NXR), and nitrite reductase (NIR). As illustrated in Fig.  3 A, 0.01 mg/g ZnO NPs exposure had no significant influence on AMO activity throughout the exposure period (*p* > 0.05). Compared with the control, AMO activity increased significantly at day 21 (Fig. 3A) for 0.5 mg/g ZnO NPs (*p* < 0.05), suggesting a concentration-dependent stimulation of ammonia oxidation at early time points., while NXR activity decreased at day 21 for 0.5 mg/g ZnO NPs (Fig. 3B), respectively. At day 42, AMO activity increased for all three concentrations of citrate-coated ZnO NPs (*p* < 0.05), while NXR activity decreased for those treatments (*p* < 0.01). After day 84, AMO activities decreased for most treatment groups while NXR activities increased. AMO catalyzes oxidation of NH_4_^+^-N to hydroxylamine (NH_2_OH) and NXR is responsible for nitrite oxidation process, catalyzing oxidation of NO_2_^−^-N to NO_3_^−^-N. The results suggest that citrate at high levels can give a short-term boost to ammonia oxidation, possibly because citrate increases ZnO solubility and Zn^2^⁺ interacts with microbial membranes, making substrates more available (Radniecki et al. 2011). However, this mechanistic interpretation is speculative as dissolved Zn^2+^ concentrations in soil pore water were not directly measured in this study. Support for citrate-enhanced ZnO dissolution comes from established chelation chemistry and studies demonstrating citrate's role in modulating metal nanoparticle dissolution kinetics (Akhil and Sudheer Khan 2017; Cardoso et al. 2021). With time, the consistent exposure creates oxidative stress, damaging AMO and reducing its activity (Yu et al. 2015). This matter because AMO controls the first step of nitrification; when it weakens, the entire nitrogen cycle slows down. Additionally, the inverse relationship observed between AMO and NXR activities suggests a feedback loop: as AMO activity increased, more nitrite was produced, but NXR was simultaneously suppressed, creating conditions for transient nitrite accumulation. Such stress–recovery cycles are common in nitrifying bacteria under nanoparticle exposure (Frenk et al. 2013; Phan et al. 2020; Wang et al. 2015).

With respect to denitrification, NIR activity remained relatively stable throughout the exposure period across all treatments (Fig. 3C). A slight increase in NIR activity was observed for citrate-coated ZnO NPs at day 63, but by day 84, there was a decline in activity, especially for the citrate-coated ZnO NPs (*p* < 0.05). Since NIR catalyzes reduction of NO_2_^−^-N to NO, its suppression under ZnO stress could lead to nitrite accumulation in soil. This imbalance in the denitrification pathway, combined with disrupted nitrification, suggests that ZnO exposure can shift the nitrogen cycle toward accumulation of intermediate nitrogen species (Ben-Moshe et al. 2013). There were no noticeable changes in HAO activity for most nanoparticle concentrations until day 63 (Fig. 3D). At day 84, there was a significant increase in HAO activity for citrate-coated ZnO NPs (*p* < 0.05) but returned to baseline by day 105. Since HAO catalyzes oxidation of NH_2_OH to NO_2_^−^-N, its rebound may indicate microbial adaptation, where certain taxa recovered this function even under nanoparticle stress (Versantvoort et al. 2020). This HAO recovery coincided with NXR activity, suggesting that upstream restoration of hydroxylamine oxidation temporarily supported downstream nitrite-to-nitrate oxidation despite overall stress.

### Impact of ZnO NPs on gene abundance

**Fig. 4:**
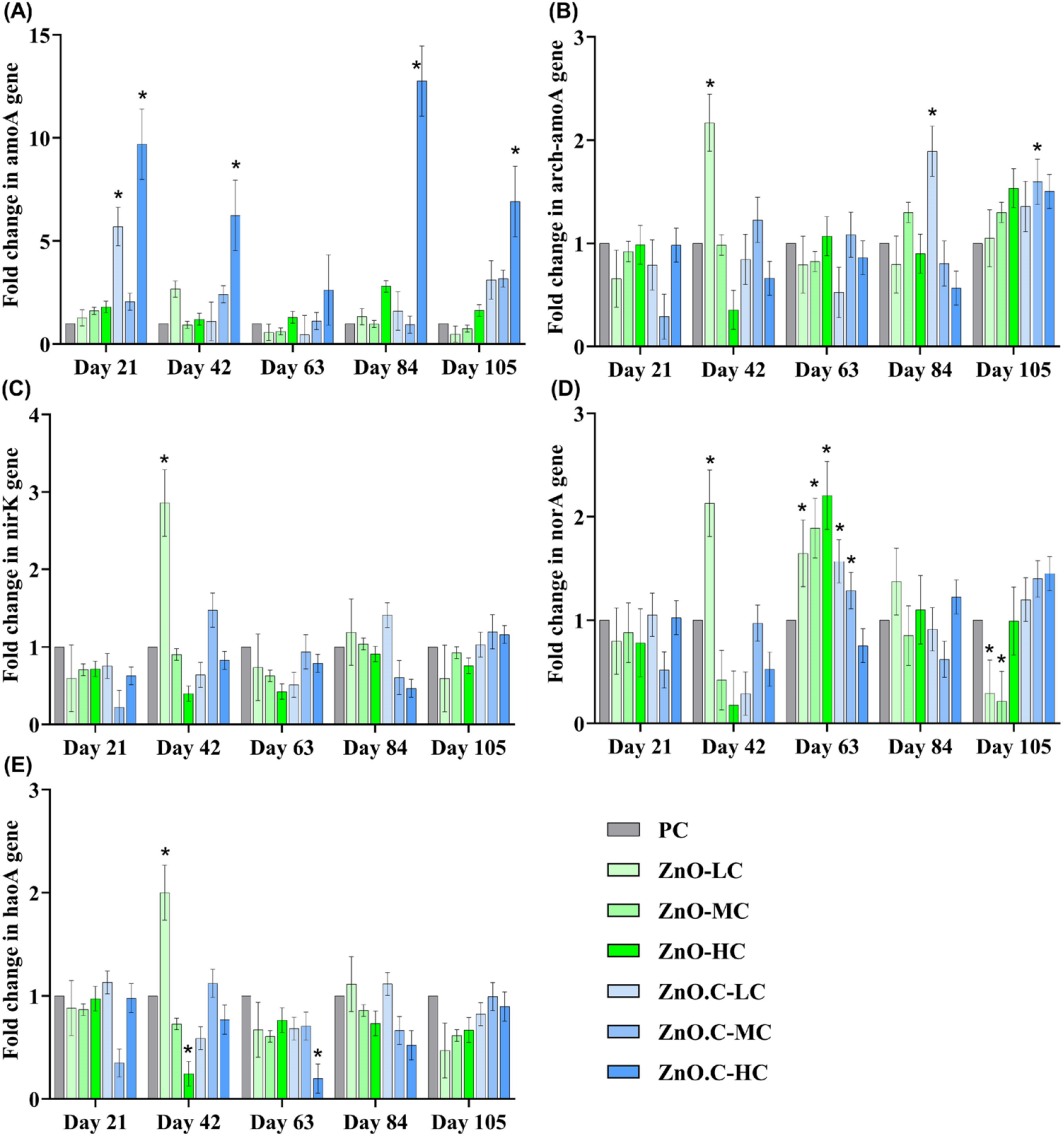
Fold change in levels of (**A**) *amoA*, (**B**) *arch-amoA*, (**C**) *nirk*, (**D**) *norA*, and (**E**) *hao* genes over time in various treatment groups. ‘PC” stands for positive control, ‘ZnO-LC’ for low concentration of ZnO NPs (0.01 mg/g of soil), ‘ZnO-MC’ for medium concentration of ZnO NPs (0.1 mg/g of soil), ‘ZnO-HC’ for high concentration of ZnO NPs (0.5 mg/g of soil), ‘ZnO.C-LC’ for low concentration of citrate-coated ZnO NPs (0.01 mg/g of soil), ‘ZnO.C-MC’ for medium concentration of citrate-coated ZnO NPs (0.1 mg/g of soil), ‘ZnO.C-HC’ for high concentration of citrate-coated ZnO NPs (0.5 mg/g of soil). Asterisks indicate statistically significant differences from the untreated control at the same time point (Kruskal–Wallis test followed by Dunn's post-hoc test, Benjamini–Hochberg FDR-corrected; *p < 0.05). Data represent mean ± SD of three independent biological replicates (*n* = 3)

The functional genes of nitrification and denitrification, including *amoA*, *arch-amoA*, *nirK*, *norA*, and *hao* were quantified and the fold change in gene abundance was calculated (Fig. 4). The fold change in *amoA* gene abundance was higher in citrate-coated ZnO NPs compared to without citrate groups (Fig. 4A). Specifically, 0.5 mg/g citrate-coated ZnO NPs significantly increased *amoA* abundance by nearly 10-fold compared with the control at days 21 and 84 (*p* < 0.05). This corresponds well with AMO enzyme activities and suggests that citrate coatings initially enhanced bacterial ammonia oxidizer populations, possibly by modulating Zn2⁺ bioavailability (Radniecki et al. 2009); though this interpretation remains speculative without direct pore water Zn^2+^ measurements. The *arch-amoA* gene remained relatively stable throughout the exposure period, with modest increases (1.5–2-fold) observed after day 63 in several treatments (Fig. 4B). In contrast to the dramatic fluctuations in bacterial *amoA*, archaeal ammonia oxidizers maintained more consistent abundances across treatments, suggesting inherent resilience to nanoparticle stress (Prosser and Nicol 2012; Yu et al. 2015). This stability rather than expansion indicates that while bacterial nitrifiers were suppressed, archaeal populations persisted and likely maintained baseline nitrification capacity. This differential response between bacterial and archaeal ammonia oxidizers likely reflects fundamental physiological differences between these two groups. Archaeal ammonia oxidizers are known to thrive under oligotrophic conditions and possess more efficient ammonia uptake systems, potentially conferring greater tolerance to metal nanoparticle stress (Prosser and Nicol 2012). In contrast, bacterial ammonia oxidizers, such as *Nitrosomonas*, are more metabolically active under nutrient-rich conditions but are also more vulnerable to membrane disruption by nanoparticles and released Zn^2+^ ions (Kapoor et al. 2015, 2018; Yu et al. 2015).

The *nirK* gene showed primarily a concentration-dependent response rather than a citrate effect (Fig. 4C). 0.01 mg/g ZnO NPs exhibited the most pronounced change, with a sharp peak at day 42 (~ 2.8-fold) followed by a decline to below baseline levels. In contrast, most other treatments, including citrate-coated ZnO NPs at all concentrations, remained relatively stable near control levels (0.5–1.5-fold range) throughout the experiment. This pattern suggests that low-concentration ZnO NPs triggered a transient denitrifier response, possibly reflecting temporary community adaptation or hormetic stimulation (Henry et al. 2004). However, the inability to sustain this response, combined with the overall suppression of denitrification enzyme activities, indicates that ZnO NP stress ultimately impaired nitrite reduction capacity. The transient spike followed by suppression could lead to nitrite accumulation in soils, as denitrifiers temporarily upregulated this function but could not maintain it under prolonged nanoparticle exposure. Similar biphasic responses have been observed with silver nanoparticles, where denitrification genes initially increased before declining (Shah et al. 2022). If this pattern occurs in field conditions, it could result in elevated soil nitrite levels and potentially increased N₂O emissions, a potent greenhouse gas. It should be noted that *nirS*, a functionally complementary denitrification gene that co-occurs with *nirK* in many soil environments, was not included in this study. Since *nirK* and *nirS*-type denitrifiers often partition across different ecological niches, the absence of *nirS* data may lead to underestimation of total denitrification potential, and this should be considered when interpreting the *nirK* results above.

The *norA* gene exhibited temporally staggered peaks across different concentration treatments (Fig. 4D). 0.01 mg/g showed elevated abundance at day 42 (~ 2.1-fold), while higher concentration treatments (both citrate and non-citrate) increased *norA* abundance at day 63 (~ 1.9–2.2 fold). By day 84–105, all treatments converged toward baseline levels, indicating recovery or adaptation. This dose- and time-dependent response pattern suggests that nitric oxide reduction was temporarily stimulated as communities responded to altered N-oxide intermediates under ZnO stress. The sequential timing of peaks at different concentrations may reflect concentration-dependent thresholds for microbial response. Unlike *nirK*, which showed a single sharp peak in LC treatment, *norA* displayed a more distributed response across multiple treatments and time points, suggesting that this step of denitrification was more broadly affected. The eventual return to baseline by day 105 across all treatments indicates that microbial communities adapted to normalize nitric oxide reduction capacity, though whether this reflects tolerance development or community restructuring cannot be determined from gene abundance alone.

The *hao* gene remained consistently low across most treatments throughout the experiment (Fig. 4E). 0.01 mg/g ZnO NPs showed a transient increase at day 42 (~ 2.0-fold), mirroring the *nirK* and *norA* patterns at this concentration and time point, but this was followed by sharp decline. All other treatments, including citrate-coated ZnO, remained near or below control levels with minimal temporal variation. Notably, citrate coating had little to no effect on *hao* gene abundance, contrasting sharply with its strong stimulation of bacterial *amoA*. The striking disconnect between low *hao* gene abundance and the observed HAO enzyme activity (particularly the rebound at day 84 for citrate-coated ZnO NPs) reveals that gene copy number does not directly predict functional activity. This mismatch can be explained by several mechanisms: (1) post-transcriptional regulation where existing *hao* transcripts are more efficiently translated under stress, (2) enzyme stability and turnover, where HAO proteins persist longer than gene abundance would suggest, or (3) functional redundancy within the microbial community, where specific taxa with constitutively high HAO activity compensate for overall low gene abundance (Zheng et al. 2024). This gene-enzyme decoupling emphasizes the importance of measuring both genetic potential and actual enzymatic function when assessing nanoparticle impacts on nitrogen cycling. It suggests that nitrification activity may be maintained through existing enzyme pools or community-level functional compensation even when the genetic capacity appears suppressed.

To formally assess gene–enzyme coupling across the dataset, Spearman rank correlations were performed between enzyme activities and corresponding functional gene fold changes across all treatments and time points (*n* = 30 per pair). No significant correlations were detected for any of the three pairs examined: AMO activity vs. bacterial *amoA* (*ρ* =  − 0.225, *p* = 0.231), HAO activity vs. *haoA* (*ρ* =  + 0.133, *p* = 0.483), or NIR activity vs. *nirK* (*ρ* =  + 0.029, *p* = 0.879). This systematic decoupling across all three enzyme–gene pairs indicates that gene copy number is not a reliable predictor of enzyme activity under ZnO NP stress conditions in this study, likely reflecting post-transcriptional regulation, differential enzyme turnover rates, functional redundancy among community members, or a combination thereof. These findings reinforce the necessity of multi-level assessments that integrate both genomic and biochemical measurements rather than relying on either in isolation.

The gene abundance data reveal three critical patterns in microbial nitrogen cycling responses to ZnO nanoparticles. First, low concentration of ZnO NPs triggered synchronized day 42 increases in *nirK*, *norA*, and *hao*, indicating a coordinated community-wide response affecting both nitrification and denitrification pathways simultaneously; likely representing a transient hormetic or adaptive response that could not be sustained under prolonged exposure. Second, citrate coating effects were highly pathway-specific: while citrate profoundly stimulated bacterial *amoA*, it had minimal impact on *arch-amoA* or downstream genes (*hao*, *nirK*, *norA*), suggesting that citrate primarily influences the initial ammonia oxidation step without cascading effects on other nitrogen-cycling guilds. Third, the striking disconnect between consistently low *hao* gene abundance and the observed HAO enzyme activity rebounds underscores that genomic potential does not directly translate to functional capacity, likely due to post-transcriptional regulation, enzyme stability, or community-level functional redundancy. These findings emphasize the necessity of multi-level assessments integrating gene abundance, enzyme activity, and process rates to comprehensively understand nanoparticle impacts on soil biogeochemical cycling.

### Impact of ZnO NPs on microbial diversity and community composition

A total of 34,287,681 sequence reads were generated for bacterial and archaeal 16S rRNA and fungal/eukaryotic 18S rRNA present in the genomic DNA of untreated and treated soil samples. The rarefaction curves derived from the total ASVs and normalized sequence reads (*n* = 173,330 sequence reads per sample) for all the samples is presented in Figure S2. A total of 23,765 amplicon sequence variants (ASVs) were detected in both untreated and treated samples, with their counts in individual samples varying between 2280 and 5677. The feature classifier categorized all ASVs from both untreated and treated samples into 43 phyla, including 7 archaea, 33 bacteria, and 3 eukaryotes, with their relative abundances differing according to the sample type.

**Fig. 5:**
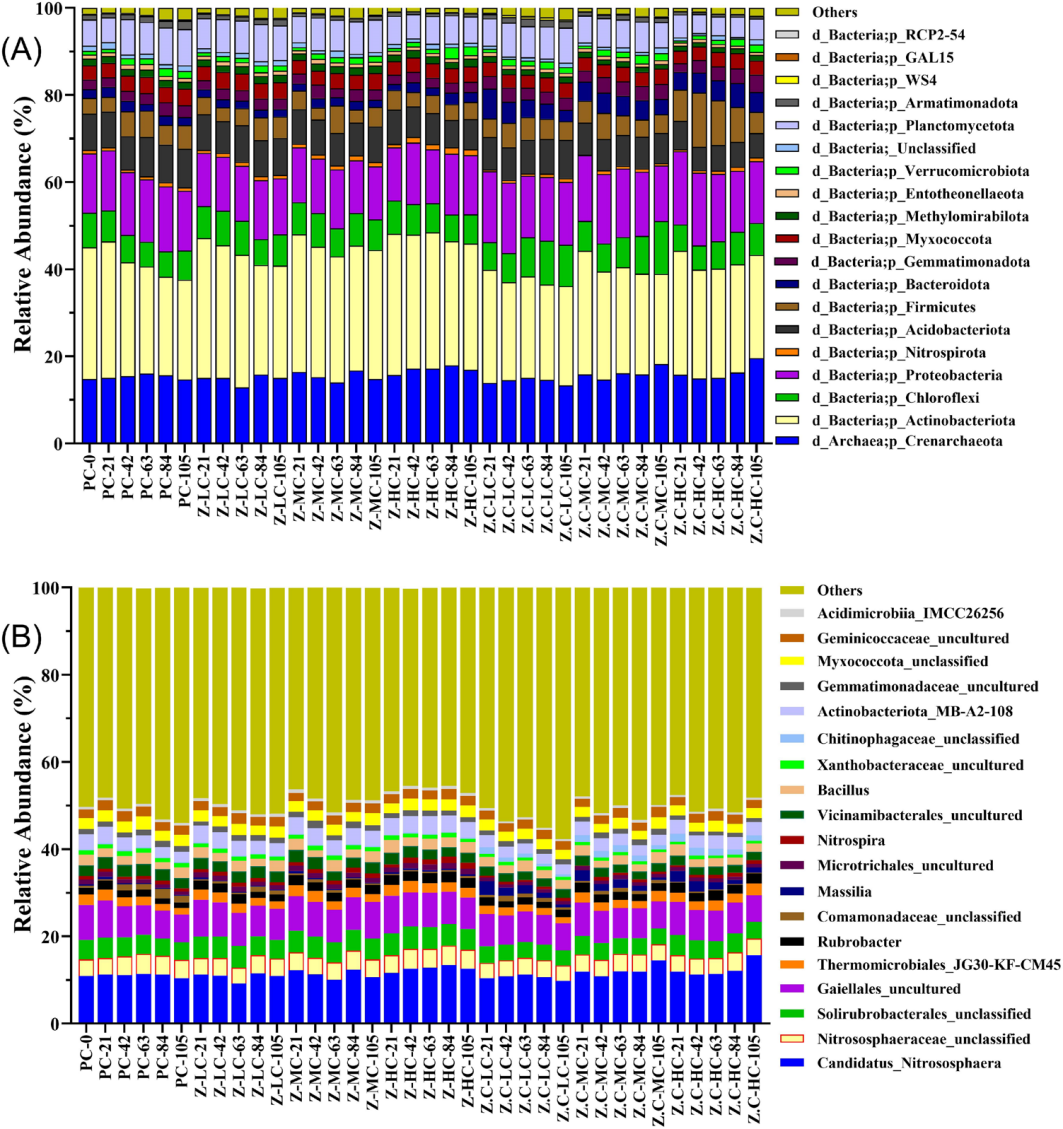
Relative abundance of the top 20 (**A**) phyla and (**B**) genera detected in the untreated and treated (ZnO and citrate-coated ZnO NPs) soil samples. ‘PC” stands for positive control, ‘Z-LC’ for low concentration of ZnO NPs (0.01 mg/g of soil), ‘Z-MC’ for medium concentration of ZnO NPs (0.1 mg/g of soil), ‘Z-HC’ for high concentration of ZnO NPs (0.5 mg/g of soil), ‘Z.C-LC’ for low concentration of citrate-coated ZnO NPs (0.01 mg/g of soil), ‘Z.C-MC’ for medium concentration of citrate-coated ZnO NPs (0.1 mg/g of soil), ‘Z.C-HC’ for high concentration of citrate-coated ZnO NPs (0.5 mg/g of soil)

The relative abundance of the top 20 identified phyla detected in soil samples is presented in Fig. 5A. While untreated and ZnO-treated soil samples showed that archaeal phylum Crenarchaeota and bacterial phyla including Actinobacteriota, Proteobacteria, and Acidobacteriota as the main phyla, representing around 60% of the overall relative abundance, the citrate-coated ZnO-treated soil samples showed archaeal phylum Crenarchaeota and bacterial phyla Actinobacteriota, Proteobacteria, and Chloroflexi are more abundant and collectively constituted approximately 90% of the total relative abundance. Figure 5B illustrates the relative abundance of the top 20 genera identified in the genomic DNA of untreated and treated soil samples, with *Nitrososphaera* (archaea) and *Gaiellales*_uncultured (bacteria) as most abundant genera. The following subsections present more details about nitrogen-metabolizing bacterial and archaeal genera, along with alpha and beta diversity assessment for the untreated and treated soil samples.

The alpha diversity indices for the microbial communities of untreated and treated soil samples, including Faith’s phylogenetic diversity, Pielou evenness, and the Shannon diversity index (H′), are presented in Figures S3A, S3B, and S3C, respectively. Additionally, the individual Shannon diversity index values for each sample are provided in Table S3. The analysis of alpha diversity demonstrated notable differences in microbial community richness and evenness across the various treatment conditions. The untreated soil samples (PC) demonstrated relatively higher values of Faith's phylogenetic diversity and Pielou’s evenness throughout the testing period, suggesting the presence of stable and diverse microbial communities. Treated soil samples demonstrated that low (0.01 mg/g) and medium (0.1 mg/g) concentrations of ZnO and citrate-coated ZnO NPs exhibited comparatively higher diversity, whereas samples treated with high (0.5 mg/g) concentrations exhibited the lowest levels of diversity and evenness, suggesting concentration-dependent inhibition. The Shannon diversity index, a robust metric for assessing species richness and evenness that is less susceptible to PCR errors (Reeder and Knight 2009), demonstrated distinct patterns in microbial community complexity across treatments from day 21 to day 105 (Table S3). The untreated soil samples (PC) demonstrated moderate variability in H′ values while exhibiting an increase in alpha diversity, concluding with the highest H′ value recorded on day 105 (H′ = 10.86). ZnO-treated samples at low and medium concentrations demonstrated consistently high microbial diversity (H′ > 10.5) throughout all time points, suggesting minimal disturbance to the microbial community structure. In contrast, the treatment with high concentrations of ZnO NPs exhibited a slight reduction and greater variability in diversity, with H′ values between 10.25 and 10.53, indicating a moderate concentration-dependent nanoparticle stress on soil microbial communities. Notably, the treatment with citrate-coated ZnO NPs showed significant effects, as the low concentration showed a steady increase in diversity reaching highest at day 105 (H′ = 10.98), while the medium concentration exhibited a decline in H′ values (9.98) at later stages, and the high concentration consistently displayed lowest Shannon index values, reaching H′ of 9.80 at day 105. While the pairwise Kruskal-Wallis analysis found no significant differences in Shannon diversity between the positive control and treatment types (across all concentrations of pristine ZnO and citrate-coated ZnO combined), the comparison of pristine ZnO and citrate-coated ZnO yielded a lower *p*-value (*p* = 0.078). When concentrations were compared, significant concentration-dependent effects were observed, with the high-concentration group differing notably from both the low-concentration (*p* = 0.0001) and medium-concentration (*p* = 0.004) treatments. These findings suggest that both the type of treatment and its concentration influence community structure, with elevated levels of citrate-coated ZnO NPs having the most significant negative effects on microbial richness and evenness.

**Fig. 6:**
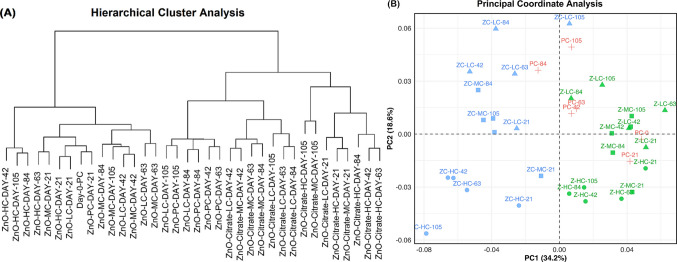
Beta diversity matrices generated for the microbial communities of the untreated and treated (ZnO and citrate-coated ZnO NPs) soil samples. (**A**) Hierarchical Cluster Analysis (HCA) and (**B**) Principal Coordinate Analysis (PCoA) plot, illustrating the differences in microbial community composition across treatments. ‘PC” stands for positive control, ‘ZnO-LC’ for low concentration of ZnO NPs (0.01 mg/g of soil), ‘ZnO-MC’ for medium concentration of ZnO NPs (0.1 mg/g of soil), ‘ZnO-HC’ for high concentration of ZnO NPs (0.5 mg/g of soil), ‘ZnO.C-LC’ for low concentration of citrate-coated ZnO NPs (0.01 mg/g of soil), ‘ZnO.C-MC’ for medium concentration of citrate-coated ZnO NPs (0.1 mg/g of soil), ‘ZnO.C-HC’ for high concentration of citrate-coated ZnO NPs (0.5 mg/g of soil)

The beta diversity indices, including HCA and PCoA, were utilized to evaluate the similarities in microbial community structure and composition between untreated and treated samples. HCA identified distinct clustering patterns in microbial communities linked to the treatments and concentrations, with untreated and ZnO-treated soil samples exhibiting closer associations, while citrate-coated ZnO NP-treated soil samples formed separate clusters, highlighting significant differences in microbial community structure (Fig. 6A). Among untreated and ZnO-treated soil samples, untreated samples and those with low to medium ZnO NP concentrations grouped closely, demonstrating similar microbial communities with minimal disruption, whereas samples treated with high ZnO NP concentrations exhibited distinct clustering, suggesting a variation in community structure. In the soil samples treated with citrate-coated ZnO NPs, samples with low and medium concentrations were grouped together, whereas high concentration samples formed a distinct cluster, suggesting that concentration influences the structure of the microbial community. The PCoA analysis revealed that PC1 (34.2%) and PC2 (18.6%) account for most of the variance in the data (Fig. 6B) and also indicated that the microbial communities in citrate-coated ZnO NP treated soil samples differ significantly from those in untreated and ZnO-treated samples, aligning with the findings of the HCA. The PERMANOVA analysis of weighted UniFrac distances indicated that the microbial community composition was significantly influenced by treatment type (*R*^2^ = 0.275, *p* = 0.001), day (*R*^2^ = 0.218, *p* = 0.001), and concentration (*R*^2^ = 0.147, *p* = 0.001). Among these, treatment type accounted for the majority of the variance, highlighting its primary influence on community structure.

Although the feature classifier identified around 700 different genera from the ASVs in untreated and ZnO and citrate-coated ZnO NP-treated soil samples, as mentioned earlier, this study primarily focused on nitrogen-metabolizing bacterial and archaeal genera. Subsequently, this study discusses the bacterial and archaeal genera associated with nitrogen fixation, ammonia oxidation, nitrite oxidation, and denitrification. The classification of these nitrogen-cycling functional microbial groups was primarily based on the taxonomic affiliation of genera reported in the literature for respective categories. A total of twelve genera related to bacteria and archaea were identified as nitrogen-fixing microorganisms (NFM), which were reported to carry nitrogen fixation genes such as *nifH*, and their overall relative abundance is illustrated in Figure S4A. The members of NFM include 11 bacterial genera (*Azoarcus, Azospirillum, Azotobacter, Bradyrhizobium, Cupriavidus, Clostridium, Mesorhizobium, Methylobacterium, Microvirga Nostoc, Paenibacillus, and Rhizobium)* along with one archaeal genus (*Methanosarcina*)*.* The total sequence reads generated for each of these genera are given in Table S4. The relative abundance of NFMs exhibited considerable variation among different treatment groups and types. Among untreated, ZnO and citrate-coated ZnO NP-treated soil samples, the NFMs were most predominant in the 0.01 mg/g citrate-coated ZnO NP samples, indicating that a low concentration of citrate-coated ZnO NP treatment can support their growth. While all treatment types of ZnO (LC, MC, and HC) soil samples showed a gradual decline in NFM abundance and were less than the untreated soil sample’s relative abundance at day 105, the 0.01 mg/g ZnO NPs and 0.5 mg/g citrate-coated ZnO NPs soil samples displayed the lowest NFMs abundance among all samples, indicating these concentrations are severely affecting nitrogen-fixing bacterial and archaeal genera.

Regarding ammonia-oxidizing microorganisms (AOM), which are key contributors to the first step of nitrification, a total of five bacterial and archaeal genera were detected, and their overall relative abundance is presented in Figure S4B. The members of AOMs include 2 bacterial (*Nitrosomonas* and *Nitrosospira*) and 3 archaeal (*Candidatus_Nitrososphaera, Candidatus_Nitrocosmicus*, and members of the *Nitrososphaeraceae* family) genera that carry ammonia-oxidation genes such as *amoA* and *arch-amoA*. While bacterial genera were lower in abundance, the archaeal genera dominated the ammonia-oxidizing microorganisms in both untreated and treated soil samples, and the total sequence reads generated for each of these genera is given in Table S5. In untreated soil samples (PC), AOMs were consistently present with no significant change in their relative abundance. While low and medium concentrations of ZnO-treated soil samples showed a relatively similar trend to that of untreated soil samples during 105 days of exposure, the high concentration of ZnO-treated soil samples showed a noticeable increase in the relative abundance of AOMs, suggesting an increase in ammonia oxidation with higher ZnO NP concentration. In the citrate-coated ZnO NP-treated soil samples, low concentration conditions resulted in a minor reduction in the relative abundance of AOMs compared to the untreated soil, whereas the medium and high concentrations at day 105 demonstrated a significant increase in AOMs, indicating active ammonia oxidation at elevated citrate-coated ZnO NP concentrations.

Regarding the nitrite-oxidizing microorganisms (NOM), which are responsible for converting nitrite to nitrate in the nitrification process, only 2 bacterial genera were detected, and their overall relative abundance is presented in Figure S4C. The members of NOM include the genera *Nitrospira* and *Nitrolancea*, which possess nitrite oxidoreductase genes (such as *nxrA* and *nxrB*) that mediate the oxidation of nitrite to nitrate (Ishii et al. 2017). The total number of sequence reads for each of these genera is listed in Table S6. Although genus *Nitrospira* has been predominantly recognized for its role as nitrite-oxidizing bacteria, it has also been reported to have comammox species harboring nitrification genes such as *amoA*, *hao*, and *nxrA/nxrB*, which enable the complete conversion of ammonia to nitrate (Daims et al. 2016). The present study identified species associated with *Nitrospira japonica* and *Nitrospira moscoviensis*, which have been documented as nitrite-oxidizing bacteria (Leung et al. 2022). In untreated soil samples (PC), the presence of NOMs remained consistent, showing no significant variation in their relative abundance, similar to that of AOMs. While low concentrations of ZnO-treated soil samples exhibited a relatively similar trend to that of untreated soil samples during 105 days of exposure, the medium and high concentration of ZnO-treated soil samples showed a noticeable increase in the relative abundance of NOMs, suggesting an increase in nitrite oxidation with an increase in ZnO concentration. In the case of the citrate-coated ZnO-treated soil samples, low and medium concentration samples showed slight reductions in the NOMs relative abundance compared to untreated soils but high concentration of citrate-coated ZnO showed significant increase in NOMs at day 105, suggesting higher nitrite oxidation only with higher citrate-coated ZnO NP concentrations.

In the case of denitrifying microorganisms (DM), a total of 15 bacterial genera were detected, and their overall relative abundance is shown in Figure S4D. The bacterial genera of DMs include *Amaricoccus, Bacillus, Bradyrhizobium, Brevibacillus, Comamonas, Dechloromonas, Fictibacillus, Flavobacterium, Lysinibacillus, Paenibacillus, Paracoccus, Pseudomonas, Rubellimicrobium, Rummeliibacillus,* and *Thiobacillus*. These genera contain key denitrification-related genes such as *nirK*, *nirS*, *norB*, and *nosZ*, which are vital for the reduction of nitrate and nitrite (Shan et al. 2021; Wei et al. 2015). The total sequence reads generated for each of these genera for the untreated and treated soil samples are given in Table S7. Among the aforementioned bacterial genera, the diazotrophic bacteria, such as *Paenibacillus* and *Bradyrhizobium*, can also denitrify (Pajares and Bohannan 2016). The untreated (PC) soil samples exhibited a relatively stable relative abundance of DM, except day 84, suggesting the presence of a consistent nitrogen-removing microbial community under these conditions. However, the relative abundance of DMs varied significantly across treatment groups (Figure S4D). In the ZnO and citrate-coated ZnO-treated samples, all three concentrations (LC, MC, and HC) of ZnO-treated soil samples maintained relatively comparable levels (although slightly lower) of DMs to those of the untreated soil samples, but all three concentrations of citrate-coated ZnO NP-treated soil samples, particularly 0.5 mg/g treated samples, exhibited notably decreased DMs, suggesting that elevated citrate-coated ZnO NPs exposure can severely disrupt the denitrification process.

ALDEx2 was used to statistically analyze the differential abundance of key microbial taxa, and results revealed that ZnO and citrate-coated ZnO nanoparticles significantly impacted the soil microbial community structure compared to the positive control (Figure S5). In general, citrate-ZnO treatments led to more significant alterations in microbial composition than ZnO treatments alone, especially under MC and HC conditions, where the highest number of notably different taxa was observed. Several genera, including *Pontibacter, Gemmatimonas, Azoarcus, Massilia, Noviherbaspirillum, Cupriavidus*, and* Dechloromonas*, showed significantly greater relative abundances when treated with citrate-ZnO, suggesting that altering nanoparticle surfaces increased selective pressure on microorganisms and changed their ecological responses.

Among the significantly enriched taxa, several diazotrophic bacteria and nitrogen-cycle-associated microorganisms were detected. *Azoarcus* and *Cupriavidus* are known diazotrophic bacteria and have been reported to possess nitrogen fixation genes (*nifH*, *nifD,* and *nifK*) (Li et al. 2012; Lindström and Mousavi 2020). The increased relative abundance of these genera under MC and HC conditions of citrate-coated ZnO NPs suggests that the treatment selectively favored NFM populations capable of nanoparticle-induced stress. However, their decline by day 105 (Table S4) indicates that extended exposure negatively impacted these bacteria. Similarly, the ammonia-oxidizing bacterium *Nitrosospira*, associated with nitrification genes such as *amoA* and *hao* (Prosser et al. 2014), showed a significant increase in abundance under 0.5 mg/g citrate-coated ZnO NPs. Extended exposure to citrate-coated ZnO NPs consistently increased *Nitrosospira* populations, indicating enhanced ammonia oxidation and supporting the development of nitrifying microbial communities. Several denitrifying microorganisms, such as *Dechloromonas* and *Pseudomonas,* that possess denitrification genes *like nirK, nirS, norB,* and *nosZ* (Shan et al. 2021)*,* showed significant enrichment under treatment with 0.1 and 0.5 mg/g citrate-coated ZnO NPs. The increase in the abundance of these specific genera under citrate-coated ZnO NP treatments suggests enhanced denitrification-related microbial processes; however, their decline at day 105 indicates that prolonged exposure could negatively affect their growth in soil ecosystems. Overall, the results show that pristine ZnO and citrate-coated ZnO NPs significantly altered the composition of soil microbial communities, with the citrate-coated nanoparticles causing even greater ecological effects. The increase in ammonia-oxidizing genera and the changes in nitrogen-fixing and denitrifying genera suggest that exposure to nanoparticles could affect various phases of soil nitrogen cycling, thus impacting nutrient transformation and ecosystem health.

This study provides a comprehensive assessment of how zinc oxide nanoparticles affect soil nitrogen cycling by integrating enzyme activities, functional gene abundances, and microbial community composition over an extended 105-day period. The results reveal complex, time-dependent responses that varied substantially with both nanoparticle concentration and surface chemistry, particularly the presence of citrate coating. Rather than simply inhibiting or stimulating microbial nitrogen cycling, ZnO nanoparticles created selective pressures that differentially affected specific functional guilds, resulting in nitrogen cycle imbalances with potential long-term ecological consequences.

There were considerable differences in the effect pristine and citrate-coated ZnO NPs on nitrogen cycling genes and enzymes. Citrate profoundly stimulated bacterial *amoA* gene abundance and corresponding AMO enzyme activity, particularly during the early-to-mid experimental period. In contrast, citrate had minimal effects on *arch-amoA*, downstream nitrification genes (*hao*), or denitrification genes (*nirK*, *norA*). This selectivity suggests that citrate's primary influence operates through modulation of Zn^2^⁺ bioavailability specifically affecting bacterial ammonia oxidizers rather than cascading through the entire nitrogen cycling network. The mechanism underlying citrate's effects likely involves its role as a chelating agent that alters ZnO nanoparticle dissolution kinetics and Zn^2^⁺ speciation in soil solution. Citrate can complex dissolved Zn^2^⁺, potentially reducing acute toxicity from free metal ions while simultaneously maintaining elevated bioavailable zinc concentrations near bacterial cell surfaces (Kapoor et al. 2015; Radniecki et al. 2009, 2011). It should be noted that cation exchange capacity (CEC) of soil was not measured in this study. Based on the soil texture and alkaline pH, CEC for comparable semi-arid sandy loam soils in south-central Texas typically ranges from 10 to 20 cmol(+)/kg (Brady and Weil 2008), suggesting moderate buffering capacity for Zn^2^⁺ adsorption. A critical limitation of this study is that we did not directly measure dissolved Zn^2^⁺ concentrations in soil pore water, which constrains our ability to definitively attribute the enhanced toxicity of citrate-coated ZnO NPs to increased Zn^2^⁺ bioavailability versus other citrate-mediated mechanisms. While our interpretation that citrate enhances ZnO dissolution and Zn^2^⁺ speciation is supported by well-established chelation chemistry and previous studies demonstrating citrate's role in modulating metal nanoparticle dissolution kinetics (Akhil and Sudheer Khan 2017; Zhang et al. 2013), the absence of pore water Zn^2^⁺ measurements introduces uncertainty into mechanistic conclusions. Additionally, the characterization data showed that citrate-coated nanoparticles had lower surface charge and better colloidal stability at neutral pH, which could enhance their transport to and interaction with bacterial cells, ultimately increasing both beneficial and toxic effects over time. The temporal pattern of bacterial *amoA* response, early stimulation followed by late-stage decline, supports a biphasic toxicity model where citrate-complexed zinc initially provides nutritional benefits or mild stress that induces adaptive responses, but chronic exposure generates cumulative oxidative stress and membrane damage that overwhelms cellular defense mechanisms (Yu et al. 2015).

In contrast to the dramatic fluctuations in bacterial ammonia oxidizers, *arch-amoA* abundance remained relatively stable throughout the experiment, with only modest increases (1.5–2-fold) observed after day 63. This stability indicates that archaeal ammonia oxidizers possess inherent stress tolerance mechanisms that allowed them to maintain population levels even as bacterial counterparts declined (Prosser and Nicol 2012). The microbial community data corroborate this finding, showing that archaeal genera, particularly *Nitrososphaeraceae*, remained consistently abundant and even increased in relative abundance at higher nanoparticle concentrations. The persistence of archaeal ammonia oxidizers has important functional implications, as they likely provided baseline nitrification capacity throughout the experiment, preventing complete functional collapse. However, the shift from bacterial-dominated to archaeal-dominated ammonia oxidation could have long-term consequences for nitrogen cycling kinetics, as bacterial ammonia oxidizers typically respond more dynamically to nutrient pulses while archaeal ammonia oxidizers provide slower, more constitutive activity (Gruber-Dorninger et al. 2015). A microbial community increasingly dependent on archaeal nitrification may exhibit reduced responsiveness to agricultural nitrogen inputs, potentially affecting fertilizer use efficiency and crop productivity.

The synchronized increase in *nirK*, *norA*, and *hao* gene abundances at day 42 specifically in the 0.01 mg/g non-citrate ZnO treatment represents one of the most intriguing findings. This coordinated response across both nitrification and denitrification pathways suggests a community-wide adaptive mechanism rather than isolated gene-specific responses. Low-dose nanoparticle exposure may trigger hormetic responses ranging from beneficial adaptations to mild stress that temporarily enhance microbial metabolic capacity, possibly through mild oxidative stress that upregulates antioxidant defenses or through micronutrient supplementation, as zinc serves as a cofactor for numerous nitrogen metabolism enzymes (Ochoa-Herrera et al. 2011). Hormesis, defined as a biphasic dose-response where low doses stimulate and high doses inhibit, has been documented in microbial systems exposed to metal nanoparticles, potentially through mild oxidative stress that upregulates antioxidant defenses or through micronutrient supplementation given zinc’s role as a cofactor for nitrogen metabolism enzymes (Agathokleous et al. 2022).

The temporal dynamics of enzyme activities reveal an imbalance in nitrogen cycling pathways under nanoparticle stress. The inverse relationship between AMO and NXR activities, early AMO stimulation with NXR suppression, followed by reversed patterns suggests disrupted coordination between sequential nitrification steps, potentially leading to transient accumulation of hydroxylamine and nitrite, both toxic intermediates. Even more concerning is the suppression of denitrification capacity, evidenced by relatively stable but low NIR activity, declining *nirK* gene abundance after early spikes, and substantially reduced denitrifying microbial populations, particularly in high citrate-coated ZnO treatments. Enzyme activities are expressed as relative activity (%) normalized to the untreated control at each time point. While this approach highlights treatment-induced changes, it precludes direct comparison with absolute activity values (e.g., nmol substrate converted g⁻^1^ dry soil h⁻^1^) reported in other studies and limits quantitative integration with absolute functional gene abundance data. Future studies should report enzyme activities in absolute units to facilitate cross-study meta-analyses and multi-level integrative assessments. The microbial community data showed that denitrifying bacteria were among the most severely affected functional groups, with citrate-coated ZnO at 0.5 mg/g causing the greatest reductions. This selective suppression creates an imbalanced nitrogen cycle where ammonia oxidation proceeds while nitrogen removal through denitrification is compromised. The ecological consequences include increased nitrate leaching into groundwater, enhanced substrate availability for N_2_O production (a potent greenhouse gas), potential nitrite toxicity to plants and soil fauna, and altered soil pH (Kuypers et al. 2018). The simultaneous suppression of nitrogen fixation further exacerbates nitrogen cycle dysfunction by reducing the input of biologically available nitrogen.

The 16S rRNA gene sequencing data revealed substantial community restructuring, particularly in citrate-coated ZnO treatments at high concentrations, with alpha diversity declining significantly and beta diversity analyses showing clear separation from controls. The differential responses of functional guilds such as severe suppression of nitrogen-fixing and denitrifying microorganisms and increased relative abundance of ammonia-oxidizing archaea and nitrite-oxidizing bacteria indicate that nanoparticles act as selective agents that restructure communities toward stress-tolerant taxa at the expense of more metabolically versatile organisms. The enrichment of specific phyla in citrate-coated ZnO treatments suggests that thick cell walls, efficient metal efflux systems, and metabolic flexibility may confer nanoparticle tolerance, though the overall reduction in diversity could decrease community resilience to additional stressors.

Several limitations of this study should be acknowledged. First, dissolved Zn^2+^ concentrations in soil pore water were not directly measured, which constrains our ability to definitively attribute the differential effects of citrate-coated ZnO NPs to increased Zn^2+^ bioavailability versus other citrate-mediated mechanisms such as altered nanoparticle dispersibility or direct carbon supplementation to the heterotrophic microbial community. Future studies should incorporate dynamic monitoring of pore water Zn^2+^ concentrations to validate the mechanistic interpretations proposed here. Second, this study did not include a soluble Zn^2+^ ion control group, which would have allowed direct differentiation between nano-specific effects and ionic effects on enzyme activities, functional gene abundances, and microbial community composition. This represents an important avenue for future investigation. Third, soil ammonium-N and nitrate–N concentrations were not monitored over the course of the experiment, which limits our ability to directly validate whether the observed changes in enzyme activities and functional gene abundances translated into measurable nitrogen transformation fluxes. Additionally, urease activity and nitrate reductase activity were not measured, leaving the mineralization (organic N → NH_4_^+^) and the initial denitrification step (NO_3_^−^ → NO_2_^−^) uncharacterized; future studies should incorporate a complete enzyme panel spanning the full mineralization–nitrification–denitrification continuum. Finally, dose-response modelling such as EC50 estimation was not performed, which limits quantitative comparison with other studies. These limitations should be addressed in future work through improved experimental designs incorporating ion controls, pore water chemistry monitoring, expanded gene panels, and dose-response modelling.

An important consideration is the substantial gap between experimental exposure concentrations (0.01–0.5 mg/g) and current environmental concentrations of ZnO nanoparticles in soils (Solymos et al. 2025; Wu 2019). However, several factors suggest that environmentally relevant concentrations could still pose risks: nanoparticles accumulate over repeated applications, may concentrate in soil microsites, and the hormetic responses observed at low concentrations suggest that even sub-toxic doses may trigger subtle community shifts. Current environmental concentrations likely do not pose immediate risks, but projected increases in nanoparticle applications could push soil concentrations toward ranges where effects manifest. The sensitivity of denitrifying bacteria and nitrogen-fixing organisms suggests these functional guilds could serve as early indicators of nanoparticle impacts, emphasizing the need for long-term field monitoring studies at realistic application rates.

## Conclusions

This comprehensive 105-day study reveals that ZnO NPs exert complex, time-dependent effects on soil nitrogen cycling that vary substantially with concentration and surface chemistry, creating selective pressures that differentially affect functional guilds and result in nitrogen cycle imbalances. Citrate coating profoundly influenced impacts in pathway-specific ways, initially stimulating bacterial ammonia oxidizers but ultimately causing more severe suppression of bacterial nitrifiers, greater reductions in microbial diversity, and more pronounced community restructuring than uncoated nanoparticles. Archaeal ammonia oxidizers demonstrated notable resilience, maintaining stable populations and providing functional compensation as bacterial nitrifiers declined, though this shift toward slower archaeal-dominated nitrogen cycling could reduce ecosystem responsiveness to fertilizer inputs. The striking disconnect between *hao* gene abundance and HAO enzyme activity emphasizes that functional capacity cannot be predicted from genetic markers alone, underscoring the necessity of multi-level assessments integrating gene quantification, enzyme activities, and process measurements. Most concerning was the creation of nitrogen cycle imbalances characterized by continued ammonia oxidation but severely impaired denitrification, which could lead to reactive nitrogen intermediate accumulation, increased nitrate leaching, enhanced N_2_O emissions, and reduced nitrogen use efficiency. While current environmental ZnO nanoparticle concentrations are orders of magnitude below experimental levels and likely pose minimal immediate risk, projected increases in agricultural nanotechnology applications and the sensitivity of key functional guilds warrant precautionary regulation and long-term monitoring.

Several limitations constrain the interpretive scope of this study and should guide future research. Dissolved Zn^2^⁺ concentrations in soil pore water were not measured, limiting our ability to attribute citrate-coated NP effects specifically to Zn^2^⁺ bioavailability rather than NP-intrinsic or carbon-mediated mechanisms; future studies should incorporate dynamic ICP-MS monitoring of pore water Zn^2^⁺. The absence of a soluble Zn^2^⁺ ionic control precludes separation of nano-specific from ionic toxicity; future designs should include molar-equivalent Zn^2^⁺ controls. Urease and nitrate reductase activities, representing the mineralization and initial denitrification steps respectively, were not assayed, leaving the broader nitrogen cycle incompletely characterized; future studies should employ a complete enzyme panel. Finally, the three-point concentration gradient used here precluded formal dose–response modeling (e.g., EC₅₀ estimation); future studies should employ concentration gradients sufficient for such modeling to enable quantitative cross-study comparison. The current findings underscore the need for extended-duration studies to capture temporal dynamics, integrated multi-level assessments, careful consideration of surface modifications that may intensify environmental impacts, and development of early warning indicators to detect subtle impacts before ecosystem-scale dysfunction occurs.

## Supplementary Information

Below is the link to the electronic supplementary material.

ESM 1 (DOCX 1.24 MB)

## Data Availability

The datasets used and/or analyzed during the current study are available from the corresponding author on reasonable request.
