# Supplementary material for: Effects of pristine and citrate-coated zinc oxide nanoparticles on soil nitrogen cycling determined using multi-level assessment of enzyme activity, functional gene abundance and microbial community composition
Source: Environ Sci Pollut Res Int. 2026 Jul 15;33(23):11875–94. doi: 10.1007/s11356-026-38042-x (PMC13424076; doi:10.1007/s11356-026-38042-x)
Supplement: Supplementary file 1 — (DOCX 1.24 MB) [file 11356_2026_38042_MOESM1_ESM.docx]

**Supplementary Information**

**Effects of pristine and citrate-coated zinc oxide nanoparticles on soil nitrogen cycling determined using multi-level assessment of enzyme activity, functional gene abundance and microbial community composition**

Ahmed Hussain^1^, Mohammad Jahid Hasan^2^, Kiran Vadde^1^, Akanksha Matta^3^, Matthew Moreno^2^, Esteban E. Ureña-Benavides^2^, Vikram Kapoor^1,2*^

^1^School of Civil & Environmental Engineering, and Construction Management, The University of Texas at San Antonio, San Antonio, TX 78249, United States of America

^2^ Department of Biomedical Engineering and Chemical Engineering, The University of Texas at San Antonio, San Antonio, TX 78249, United States of America

^3^ Department of Chemistry, The University of Texas at San Antonio, San Antonio, TX 78249, United States of America

*Corresponding author: vikram.kapoor@utsa.edu

**Synthesis of ZnO and citrate-coated ZnO nanoparticles:** Zinc acetylacetonate, Zn(acac)_2_, (≥ 99.5%), 1,4-butanediol (≥ 99.0%), and ethyl alcohol (≥ 99.5%) were purchased from Sigma-Aldrich (St. Louis, MO, USA), and were used as received. ZnO NPs were synthesized in-house via the hydrolysis of Zinc (II) acetylacetonate [Zn(acac)₂] in 1,4-butanediol at 140°C, following the method described by Visinescu et al. [1] Briefly, 9.885 g (0.25 mol/L) of Zn(acac)₂ was dissolved in 150 mL of 1,4-butanediol. The solution was heated to 140°C while stirring with a magnetic stirrer at 800 rpm in a round-bottom (RB) flask equipped with a condenser for reflux. The reaction was allowed to proceed for 5 hours, with ZnO precipitation occurring after approximately 30 minutes. After cooling to room temperature, the reaction mixture was centrifuged at 10,000 rpm for 1 hour to collect the solid particles. The solid phase was washed first with ethanol and centrifuged again under the same conditions. The wash was then repeated with water, followed by another round of centrifugation. Finally, the washed solid particles were redispersed in water, sonicated for 5 minutes using a probe sonicator, and stored in a refrigerator at 4°C in a storage bottle. Similarly, citrate-coated ZnO NPs were synthesized at citrate/ZnO molar ratios of 0.25, 0.5, and 0.8 using 2.757 g, 5.514 g, and 8.823 g of sodium citrate dihydrate, respectively, during the reaction. The rest of the procedure remained unchanged.

**Transmission Electron Microscopy:** Microscopic images of the NPs were captured using a JEOL JEM-2010F High-Resolution Transmission Electron Microscope (Nanolab Technology, Milpitas, CA). A dilute suspension (0.01 wt%) of each sample was prepared, and one drop was placed onto a Lacey Carbon 300 mesh copper TEM grid. After 2 minutes, the excess water was removed by placing filter paper on the sample to soak up the remaining liquid. The dried TEM grids were then loaded into the microscope, and images were acquired.

**Raman Spectroscopy**: Raman spectra of freeze-dried ZnO and citrate-coated ZnO NPs powder were obtained using a Horiba LabRam HR-Evolution (Piscataway, NJ, USA), equipped with a 600 grooves/mm grating, 532 nm laser excitation, CCD camera detection, and an MPlan N 100×/0.9 objective from Olympus (Waltham, MA, USA). The spectra were recorded with an acquisition time of 5 seconds and 100 accumulations to improve the signal-to-noise ratio. Before collecting the spectra, freeze-dried NPs were obtained by drying ZnO NPs suspension in a freeze-drier.

**XRD Analysis:** Powder X-ray diffractograms (XRD) were collected using Malvern Panalytical Empyrean Nano Edition (Westborough, MA, USA). The NPs suspensions were freeze-dried, loaded in a sample holder, and exposed to X-ray scans from 2θ angles of 20° to 80° at a step time of 0.7 s/step (0.03°/step) and sample revolution of 4 rev/min at 25 °C with Cu Kα radiation (λ = 0.1540 nm).

**Size and Zeta potential:** The zeta potential and mean hydrodynamic diameter of ZnO and citrate-coated ZnO NPs dispersions in water at various pH levels were measured using a Malvern Zetasizer Nano ZS (Westborough, MA, USA). Aqueous samples were prepared at concentrations of 0.1 wt% (1 mg/mL) for zeta potential measurements and 0.01 wt% (0.1 mg/mL) for dynamic light scattering (DLS) analysis. For the aging study, DLS measurements were conducted at 0, 3, and 6 hours.

**Enzyme Activity Assays**: To determine the effect of ZnO nanoparticles on nitrogen transformation at the protein level, key enzyme activities involved in nitrogen cycling were measured at designated intervals. These included ammonia monooxygenase (AMO), hydroxylamine dehydrogenase (HAO), nitrite oxidoreductase (NXR), and nitrite reductase (NIR). All enzyme activity assays were performed in triplicate, maintaining consistent incubation conditions to ensure reproducibility.

***Ammonia Monooxygenase (AMO) and Nitrite Oxidoreductase (NXR) Activity Assays***: The activities of AMO and NXR were assessed through the oxidation of ammonium to nitrite and nitrite to nitrate, respectively, following protocols based on previous studies with minor modifications (Chen et al., 2019; Zheng et al., 2011). For sample preparation, 1 g of soil sample was added to a 50 mL centrifuge tube. For AMO activity determination, 5 mL of 0.01 M phosphate-buffered saline (PBS) containing 1 mM (NH₄)₂SO₄ and 10 mM KClO₃ (pH 7.4) was added to the tube. For NXR activity, 5 mL of 0.01 M PBS containing 1 mM NaNO₂ (pH 7.4) was used instead. The centrifuge tubes were then shaken at 200 r/min for 2 hours in a water bath at 37°C in the dark. After incubation, 5 mL of 2 M KCl solution was added to extract nitrite, and the mixture was shaken at 200 r/min for 15 minutes, followed by centrifugation at 4000 rpm for 10 minutes. AMO activity was measured by the increase in nitrite concentration in the supernatant, while NXR activity was determined by the decrease in nitrite levels, both measured spectrophotometrically at 540 nm.

***Nitrite Reductase (NIR) Activity Assay***: The activity of NIR was determined under anaerobic conditions by measuring the reduction of nitrite to nitric oxide (Chen et al., 2019). For this assay, 1 g of soil sample was placed in a 10 mL anaerobic tube, and 5 mL of 0.01 M PBS containing 2 mM NaNO₂ and 15 mM glucose (pH 7.4) was added. To establish anaerobic conditions, dissolved oxygen in the PBS was reduced to below 0.5 mg/L by purging nitrogen gas into the anaerobic tubes before the assay. The tubes were then shaken at 200 r/min for 2 hours in a water bath at 37°C in the dark. Following incubation, 5 mL of 2 M KCl solution was added to extract nitrite, and the mixture was shaken for 15 minutes before centrifugation at 4000 rpm for 10 minutes. NIR activity was assessed by measuring the reduction in nitrite concentration at 540 nm spectrophotometrically.

***Hydroxylamine Dehydrogenase (HAO) Activity Assay***: HAO activity was determined by measuring the oxidation of hydroxylamine, following the method described by Zhao et al. (2012). For sample preparation, 1 g of soil sample was added to a 50 mL centrifuge tube containing 10 mL of 0.01 M Tris-HCl buffer (pH 7.1) with 1 mM NH₂OH·HCl. The tubes were shaken at 200 r/min for 2 hours in a water bath at 37°C in the dark. HAO activity was determined by measuring the disappearance of hydroxylamine in the reaction mixture spectrophotometrically.

**Table S1**. qPCR primers used in this study.

| **Target Gene** | **Primer** | **Sequence (5' → 3')** | **Reference** |
| --- | --- | --- | --- |
| *amoA* | Arch-amoAF | STAATGGTCTGGCTTAGACG | Francis et al., 2005 |
|  | Arch-amoAR | GCGGCCATCCATCTGTATGT |  |
|  | amoA1F | GGGGTTTCTACTGGTGGT | Rotthauwe et al., 1997 |
|  | amoA1R | CCCCTCKGSAAAGCCTTCTTC |  |
| *nirK* | nirK-F1aCu | ATCATGGTSCTGCCGCG | Henry et al., 2004 |
|  | nirK-R3Cu | GCCTCGATCAGRTTRTGGTT |  |
| *norA* | F1norA | CAGACCGACGTGTGCGAAAG | Zumft, 1997 |
|  | R1norA | TCYACAAGGAACGGAAGGTC |  |
| *hao* | HAO1FQ | TGAGCCAGTCCAACGTGCAT | Zhao et al., 2017 |
|  | HAO1RQ | AAGGCAACAACCCTGCCTCA |  |
| *16S rRNA* | *515F-Y* | AGAGTTTGATCCTGGCTCAG | Parada et al., 2016 |
|  | *926R-Y* | GGTTACCTTGTTACGACTT |  |

**Table S2.** Shapiro-Wilk test for normality of residuals.

| **Assay** | **n (residuals)** | **W** | **p-value** |
| --- | --- | --- | --- |
| AMO enzyme | 90 | 0.740 | <0.0001 |
| NXR enzyme | 90 | 0.788 | 0.057 |
| NIR enzyme | 90 | 0.783 | 0.030 |
| HAO enzyme | 90 | 0.933 | 0.00017 |
| amoA gene | 90 | 0.962 | 0.035 |
| Arch-amoA gene | 90 | 0.941 | 0.098 |
| norA gene | 90 | 0.932 | 0.056 |
| nirK gene | 90 | 0.952 | 0.019 |
| hao gene | 90 | 0.950 | 0.017 |

**Table S3.** Shannon diversity indices generated for the microbial communities of untreated and treated (ZnO and citrate-coated ZnO NPs) soil samples.

| **Sample Type** | **Shannon (H) Index** |
| --- | --- |
| PC-Day-0 | 10.60 |
| PC-DAY-21 | 10.25 |
| PC-DAY-42 | 10.47 |
| PC-DAY-63 | 9.95 |
| PC-DAY-84 | 10.75 |
| **PC-DAY-105** | **10.86** |
| ZnO-LC-DAY-21 | 10.72 |
| ZnO-LC-DAY-42 | 10.74 |
| ZnO-LC-DAY-63 | 10.90 |
| ZnO-LC-DAY-84 | 10.77 |
| **ZnO-LC-DAY-105** | **10.80** |
| ZnO-MC-DAY-21 | 10.58 |
| ZnO-MC-DAY-42 | 10.54 |
| ZnO-MC-DAY-63 | 10.65 |
| ZnO-MC-DAY-84 | 10.64 |
| **ZnO-MC-DAY-105** | **10.83** |
| ZnO-HC-DAY-21 | 10.51 |
| ZnO-HC-DAY-42 | 10.25 |
| ZnO-HC-DAY-63 | 10.53 |
| ZnO-HC-DAY-84 | 10.31 |
| **ZnO-HC-DAY-105** | **10.51** |
| ZnO-Citrate-LC-DAY-21 | 10.55 |
| ZnO-Citrate-LC-DAY-42 | 10.58 |
| ZnO-Citrate-LC-DAY-63 | 10.60 |
| ZnO-Citrate-LC-DAY-84 | 10.61 |
| **ZnO-Citrate-LC-DAY-105** | **10.98** |
| ZnO-Citrate-MC-DAY-21 | 10.58 |
| ZnO-Citrate-MC-DAY-42 | 10.65 |
| ZnO-Citrate-MC-DAY-63 | 10.49 |
| ZnO-Citrate-MC-DAY-84 | 10.74 |
| **ZnO-Citrate-MC-DAY-105** | **9.98** |
| ZnO-Citrate-HC-DAY-21 | 10.31 |
| ZnO-Citrate-HC-DAY-42 | 10.01 |
| ZnO-Citrate-HC-DAY-63 | 10.10 |
| ZnO-Citrate-HC-DAY-84 | 10.19 |
| **ZnO-Citrate-HC-DAY-105** | **9.80** |

**Table S4**. Sequence reads assigned to NFM related bacterial/archaeal genera detected in the untreated and treated soil samples.

|  | ***Nostoc*** | ***Azotobacter*** | ***Clostridium*** | ***Cupriavidus*** | ***Methylobacterium*** | ***Microvirga*** | ***Paenibacillus*** | ***Bradyrhizobium*** | ***Mesorhizobium*** | ***Rhizobium*** | ***Azospirillum*** | ***Azoarcus*** | ***Methanosarcina*** |
| --- | --- | --- | --- | --- | --- | --- | --- | --- | --- | --- | --- | --- | --- |
| **PC-0** | 0 | 0 | 46 | 0 | 10 | 1175 | 390 | 227 | 84 | 70 | 14 | 0 | 0 |
| **PC-21** | 0 | 0 | 22 | 0 | 0 | 857 | 327 | 221 | 85 | 40 | 0 | 0 | 0 |
| **PC-42** | 0 | 0 | 46 | 0 | 45 | 1133 | 432 | 282 | 90 | 80 | 0 | 18 | 0 |
| **PC-63** | 0 | 0 | 28 | 0 | 0 | 531 | 256 | 152 | 22 | 27 | 13 | 5 | 0 |
| **PC-84** | 0 | 0 | 39 | 0 | 0 | 1156 | 498 | 354 | 115 | 72 | 17 | 12 | 0 |
| **PC-105** | 0 | 0 | 91 | 0 | 0 | 1420 | 756 | 383 | 83 | 68 | 18 | 20 | 0 |
| **ZnO-LC-21** | 0 | 0 | 66 | 8 | 0 | 1511 | 789 | 406 | 121 | 27 | 8 | 9 | 4 |
| **ZnO-LC-42** | 0 | 0 | 54 | 0 | 10 | 1239 | 462 | 393 | 100 | 81 | 9 | 12 | 0 |
| **ZnO-LC-63** | 0 | 0 | 31 | 0 | 12 | 1231 | 652 | 387 | 55 | 41 | 17 | 0 | 0 |
| **ZnO-LC-84** | 0 | 0 | 43 | 0 | 6 | 1236 | 573 | 408 | 97 | 55 | 0 | 0 | 4 |
| **ZnO-LC-105** | 0 | 0 | 91 | 0 | 5 | 1028 | 646 | 326 | 95 | 57 | 0 | 15 | 0 |
| **ZnO-MC-21** | 0 | 0 | 92 | 26 | 9 | 1369 | 735 | 275 | 120 | 33 | 0 | 22 | 5 |
| **ZnO-MC-42** | 0 | 0 | 36 | 8 | 6 | 970 | 329 | 267 | 76 | 17 | 0 | 0 | 3 |
| **ZnO-MC-63** | 0 | 0 | 238 | 0 | 7 | 963 | 604 | 403 | 82 | 56 | 343 | 8 | 0 |
| **ZnO-MC-84** | 0 | 0 | 37 | 9 | 0 | 1066 | 456 | 321 | 13 | 37 | 0 | 0 | 0 |
| **ZnO-MC-105** | 0 | 16 | 118 | 0 | 0 | 1477 | 905 | 477 | 168 | 47 | 22 | 16 | 0 |
| **ZnO-HC-21** | 0 | 0 | 40 | 0 | 0 | 1216 | 577 | 280 | 103 | 43 | 0 | 0 | 4 |
| **ZnO-HC-42** | 0 | 7 | 32 | 62 | 19 | 1031 | 434 | 268 | 62 | 18 | 0 | 90 | 0 |
| **ZnO-HC-63** | 0 | 0 | 73 | 97 | 0 | 1189 | 643 | 317 | 119 | 34 | 9 | 99 | 0 |
| **ZnO-HC-84** | 0 | 10 | 56 | 81 | 0 | 1169 | 448 | 332 | 33 | 35 | 15 | 32 | 0 |
| **ZnO-HC-105** | 0 | 0 | 53 | 110 | 0 | 1371 | 669 | 345 | 87 | 27 | 21 | 97 | 5 |
| **ZnO.C-LC-21** | 0 | 0 | 63 | 12 | 9 | 2231 | 614 | 302 | 81 | 57 | 0 | 193 | 0 |
| **ZnO.C-LC-42** | 0 | 0 | 12 | 0 | 5 | 1564 | 427 | 276 | 65 | 34 | 21 | 150 | 5 |
| **ZnO.C-LC-63** | 0 | 0 | 73 | 7 | 0 | 1634 | 558 | 248 | 98 | 24 | 36 | 130 | 8 |
| **ZnO.C-LC-84** | 0 | 0 | 47 | 0 | 0 | 1366 | 385 | 229 | 19 | 23 | 34 | 99 | 0 |
| **ZnO.C-LC-105** | 70 | 0 | 77 | 0 | 7 | 1806 | 558 | 372 | 94 | 30 | 75 | 61 | 0 |
| **ZnO.C-MC-21** | 0 | 0 | 60 | 38 | 16 | 1903 | 667 | 276 | 82 | 61 | 17 | 134 | 0 |
| **ZnO.C-MC-42** | 0 | 0 | 31 | 23 | 9 | 1762 | 407 | 310 | 38 | 57 | 27 | 101 | 3 |
| **ZnO.C-MC-63** | 0 | 0 | 47 | 18 | 0 | 1491 | 527 | 266 | 92 | 39 | 21 | 132 | 0 |
| **ZnO.C-MC-84** | 0 | 0 | 27 | 43 | 6 | 1645 | 475 | 390 | 82 | 26 | 25 | 157 | 0 |
| **ZnO.C-MC-105** | 0 | 0 | 20 | 18 | 0 | 555 | 209 | 91 | 0 | 0 | 17 | 101 | 0 |
| **ZnO.C-HC-21** | 0 | 0 | 209 | 640 | 34 | 1556 | 596 | 192 | 67 | 42 | 13 | 273 | 0 |
| **ZnO.C-HC-42** | 0 | 0 | 163 | 763 | 0 | 955 | 212 | 169 | 21 | 35 | 0 | 174 | 0 |
| **ZnO.C-HC-63** | 0 | 0 | 246 | 701 | 0 | 792 | 544 | 210 | 0 | 64 | 0 | 202 | 0 |
| **ZnO.C-HC-84** | 0 | 0 | 68 | 547 | 0 | 709 | 328 | 207 | 0 | 0 | 11 | 62 | 0 |
| **ZnO.C-HC-105** | 0 | 0 | 32 | 302 | 0 | 513 | 265 | 96 | 0 | 0 | 14 | 126 | 5 |

**Table S5.** Sequence reads assigned to AOM related bacterial/archaeal genera detected in the untreated and treated soil samples.

|  | ***Nitrosomonas*** | ***Nitrosospira*** | ***Candidatus_Nitrososphaera*** | ***Nitrososphaeraceae*** | ***Candidatus_Nitrocosmicus*** |
| --- | --- | --- | --- | --- | --- |
| **PC-0** | 0 | 0 | 17106 | 6017 | 115 |
| **PC-21** | 0 | 12 | 12870 | 4302 | 97 |
| **PC-42** | 24 | 8 | 19074 | 7480 | 104 |
| **PC-63** | 34 | 0 | 9522 | 3872 | 39 |
| **PC-84** | 106 | 0 | 27250 | 10499 | 131 |
| **PC-105** | 100 | 21 | 24816 | 9968 | 118 |
| **ZnO-LC-21** | 0 | 18 | 25602 | 8605 | 117 |
| **ZnO-LC-42** | 8 | 12 | 21698 | 7884 | 109 |
| **ZnO-LC-63** | 9 | 13 | 19404 | 7556 | 93 |
| **ZnO-LC-84** | 63 | 10 | 27052 | 9913 | 134 |
| **ZnO-LC-105** | 42 | 5 | 23389 | 8646 | 130 |
| **ZnO-MC-21** | 8 | 19 | 25505 | 8607 | 104 |
| **ZnO-MC-42** | 7 | 8 | 19089 | 6438 | 95 |
| **ZnO-MC-63** | 39 | 13 | 17390 | 6915 | 120 |
| **ZnO-MC-84** | 15 | 9 | 23245 | 8053 | 111 |
| **ZnO-MC-105** | 25 | 10 | 28110 | 10965 | 106 |
| **ZnO-HC-21** | 4 | 12 | 20355 | 7186 | 119 |
| **ZnO-HC-42** | 69 | 38 | 20439 | 7330 | 98 |
| **ZnO-HC-63** | 53 | 40 | 28060 | 9543 | 123 |
| **ZnO-HC-84** | 84 | 36 | 26623 | 8854 | 76 |
| **ZnO-HC-105** | 143 | 45 | 30040 | 10247 | 88 |
| **ZnO.C-LC-21** | 18 | 11 | 24499 | 8163 | 122 |
| **ZnO.C-LC-42** | 40 | 10 | 19982 | 6881 | 106 |
| **ZnO.C-LC-63** | 41 | 15 | 24665 | 8506 | 149 |
| **ZnO.C-LC-84** | 50 | 8 | 19841 | 7331 | 128 |
| **ZnO.C-LC-105** | 35 | 104 | 19705 | 6977 | 147 |
| **ZnO.C-MC-21** | 31 | 18 | 26277 | 8575 | 168 |
| **ZnO.C-MC-42** | 55 | 18 | 23369 | 8124 | 184 |
| **ZnO.C-MC-63** | 61 | 27 | 22711 | 7518 | 119 |
| **ZnO.C-MC-84** | 66 | 33 | 28905 | 9509 | 115 |
| **ZnO.C-MC-105** | 20 | 33 | 14587 | 3664 | 27 |
| **ZnO.C-HC-21** | 14 | 40 | 24258 | 7680 | 118 |
| **ZnO.C-HC-42** | 15 | 74 | 16497 | 5453 | 57 |
| **ZnO.C-HC-63** | 47 | 110 | 19720 | 6313 | 90 |
| **ZnO.C-HC-84** | 0 | 278 | 22662 | 7817 | 89 |
| **ZnO.C-HC-105** | 46 | 123 | 17309 | 4186 | 44 |

**Table S6.** Sequence reads assigned to NOM related bacterial/archaeal genera detected in the untreated and treated soil samples.

|  | ***Nitrospira*** | ***Nitrolancea*** |
| --- | --- | --- |
| **PC-0** | 1260 | 89 |
| **PC-21** | 772 | 70 |
| **PC-42** | 987 | 87 |
| **PC-63** | 579 | 86 |
| **PC-84** | 2183 | 170 |
| **PC-105** | 1585 | 165 |
| **ZnO-LC-21** | 1499 | 138 |
| **ZnO-LC-42** | 1695 | 141 |
| **ZnO-LC-63** | 1883 | 131 |
| **ZnO-LC-84** | 2139 | 150 |
| **ZnO-LC-105** | 1592 | 169 |
| **ZnO-MC-21** | 1754 | 129 |
| **ZnO-MC-42** | 1584 | 139 |
| **ZnO-MC-63** | 1543 | 83 |
| **ZnO-MC-84** | 2131 | 104 |
| **ZnO-MC-105** | 2518 | 147 |
| **ZnO-HC-21** | 1312 | 138 |
| **ZnO-HC-42** | 2051 | 129 |
| **ZnO-HC-63** | 2471 | 159 |
| **ZnO-HC-84** | 2906 | 169 |
| **ZnO-HC-105** | 3244 | 139 |
| **ZnO.C-LC-21** | 1022 | 111 |
| **ZnO.C-LC-42** | 1062 | 102 |
| **ZnO.C-LC-63** | 981 | 148 |
| **ZnO.C-LC-84** | 1134 | 97 |
| **ZnO.C-LC-105** | 1393 | 120 |
| **ZnO.C-MC-21** | 302 | 122 |
| **ZnO.C-MC-42** | 1787 | 104 |
| **ZnO.C-MC-63** | 1066 | 123 |
| **ZnO.C-MC-84** | 1800 | 138 |
| **ZnO.C-MC-105** | 467 | 91 |
| **ZnO.C-HC-21** | 870 | 126 |
| **ZnO.C-HC-42** | 914 | 104 |
| **ZnO.C-HC-63** | 1310 | 112 |
| **ZnO.C-HC-84** | 1728 | 134 |
| **ZnO.C-HC-105** | 946 | 135 |

**Table S7.** Sequence reads assigned to DM related bacterial/archaeal genera detected in the untreated and treated soil samples.

|  | ***Amaricoccus*** | ***Bacillus*** | ***Bradyrhizobium*** | ***Brevibacillus*** | ***Comamonas*** | ***Dechloromonas*** | ***Fictibacillus*** | ***Flavobacterium*** |
| --- | --- | --- | --- | --- | --- | --- | --- | --- |
| **PC-0** | 15 | 3709 | 227 | 82 | 5 | 0 | 72 | 3 |
| **PC-21** | 6 | 2343 | 221 | 32 | 16 | 0 | 51 | 0 |
| **PC-42** | 15 | 3453 | 282 | 61 | 8 | 0 | 51 | 0 |
| **PC-63** | 0 | 2153 | 152 | 69 | 0 | 0 | 51 | 0 |
| **PC-84** | 0 | 3830 | 354 | 34 | 1 | 0 | 77 | 0 |
| **PC-105** | 18 | 5753 | 383 | 149 | 8 | 0 | 124 | 0 |
| **ZnO-LC-21** | 16 | 5465 | 406 | 121 | 9 | 0 | 104 | 0 |
| **ZnO-LC-42** | 11 | 3677 | 393 | 85 | 2 | 0 | 62 | 0 |
| **ZnO-LC-63** | 8 | 4965 | 387 | 55 | 2 | 0 | 90 | 0 |
| **ZnO-LC-84** | 26 | 4092 | 408 | 79 | 13 | 0 | 88 | 0 |
| **ZnO-LC-105** | 32 | 5301 | 326 | 79 | 2 | 0 | 80 | 0 |
| **ZnO-MC-21** | 16 | 5452 | 275 | 132 | 0 | 0 | 118 | 11 |
| **ZnO-MC-42** | 19 | 2482 | 267 | 42 | 20 | 0 | 64 | 4 |
| **ZnO-MC-63** | 22 | 4485 | 403 | 163 | 0 | 0 | 74 | 0 |
| **ZnO-MC-84** | 16 | 3392 | 321 | 54 | 0 | 0 | 61 | 0 |
| **ZnO-MC-105** | 13 | 6539 | 477 | 122 | 2 | 0 | 140 | 0 |
| **ZnO-HC-21** | 0 | 5020 | 280 | 129 | 15 | 0 | 103 | 0 |
| **ZnO-HC-42** | 10 | 3300 | 268 | 59 | 0 | 58 | 64 | 0 |
| **ZnO-HC-63** | 11 | 5416 | 317 | 128 | 12 | 26 | 99 | 0 |
| **ZnO-HC-84** | 9 | 4448 | 332 | 41 | 0 | 43 | 98 | 0 |
| **ZnO-HC-105** | 10 | 5658 | 345 | 177 | 0 | 23 | 96 | 0 |
| **ZnO.C-LC-21** | 28 | 5074 | 302 | 109 | 0 | 0 | 89 | 2 |
| **ZnO.C-LC-42** | 13 | 3303 | 276 | 73 | 15 | 0 | 65 | 0 |
| **ZnO.C-LC-63** | 14 | 5565 | 248 | 91 | 0 | 0 | 88 | 2 |
| **ZnO.C-LC-84** | 10 | 3478 | 229 | 31 | 8 | 0 | 69 | 0 |
| **ZnO.C-LC-105** | 19 | 4074 | 372 | 101 | 7 | 0 | 81 | 8 |
| **ZnO.C-MC-21** | 20 | 5329 | 276 | 90 | 16 | 10 | 102 | 0 |
| **ZnO.C-MC-42** | 17 | 3570 | 310 | 102 | 0 | 38 | 70 | 0 |
| **ZnO.C-MC-63** | 16 | 3995 | 266 | 97 | 10 | 14 | 71 | 0 |
| **ZnO.C-MC-84** | 12 | 3924 | 390 | 47 | 19 | 31 | 72 | 0 |
| **ZnO.C-MC-105** | 0 | 2501 | 91 | 9 | 9 | 10 | 23 | 0 |
| **ZnO.C-HC-21** | 16 | 5200 | 192 | 99 | 5 | 204 | 85 | 3 |
| **ZnO.C-HC-42** | 0 | 2581 | 169 | 0 | 0 | 236 | 47 | 0 |
| **ZnO.C-HC-63** | 0 | 3344 | 210 | 71 | 0 | 333 | 50 | 0 |
| **ZnO.C-HC-84** | 0 | 3187 | 207 | 47 | 0 | 273 | 84 | 0 |
| **ZnO.C-HC-105** | 0 | 2139 | 96 | 28 | 0 | 79 | 21 | 0 |

**Cont. Table S7**. Sequence reads assigned to DM related bacterial/archaeal genera detected in the untreated and treated soil samples.

|  | ***Lysinibacillus*** | ***Paenibacillus*** | ***Paracoccus*** | ***Pseudomonas*** | ***Rubellimicrobium*** | ***Rummeliibacillus*** | ***Thiobacillus*** |
| --- | --- | --- | --- | --- | --- | --- | --- |
| **PC-0** | 147 | 390 | 15 | 0 | 65 | 0 | 0 |
| **PC-21** | 86 | 327 | 0 | 8 | 37 | 0 | 0 |
| **PC-42** | 202 | 432 | 0 | 12 | 54 | 0 | 0 |
| **PC-63** | 72 | 256 | 0 | 0 | 39 | 0 | 0 |
| **PC-84** | 174 | 498 | 0 | 0 | 47 | 0 | 0 |
| **PC-105** | 260 | 756 | 0 | 0 | 88 | 9 | 0 |
| **ZnO-LC-21** | 201 | 789 | 0 | 0 | 88 | 0 | 0 |
| **ZnO-LC-42** | 153 | 462 | 0 | 0 | 58 | 0 | 0 |
| **ZnO-LC-63** | 234 | 652 | 0 | 0 | 47 | 8 | 0 |
| **ZnO-LC-84** | 169 | 573 | 0 | 0 | 52 | 0 | 0 |
| **ZnO-LC-105** | 185 | 646 | 0 | 0 | 54 | 0 | 0 |
| **ZnO-MC-21** | 315 | 735 | 0 | 11 | 66 | 0 | 0 |
| **ZnO-MC-42** | 90 | 329 | 0 | 4 | 51 | 0 | 0 |
| **ZnO-MC-63** | 223 | 604 | 0 | 22 | 63 | 8 | 0 |
| **ZnO-MC-84** | 210 | 456 | 0 | 0 | 7 | 0 | 0 |
| **ZnO-MC-105** | 296 | 905 | 0 | 27 | 61 | 0 | 0 |
| **ZnO-HC-21** | 209 | 577 | 0 | 7 | 81 | 0 | 0 |
| **ZnO-HC-42** | 118 | 434 | 0 | 18 | 58 | 0 | 0 |
| **ZnO-HC-63** | 248 | 643 | 0 | 0 | 45 | 8 | 0 |
| **ZnO-HC-84** | 165 | 448 | 0 | 7 | 47 | 0 | 0 |
| **ZnO-HC-105** | 235 | 669 | 0 | 0 | 29 | 10 | 0 |
| **ZnO.C-LC-21** | 228 | 614 | 0 | 0 | 258 | 0 | 0 |
| **ZnO.C-LC-42** | 122 | 427 | 0 | 0 | 186 | 0 | 0 |
| **ZnO.C-LC-63** | 182 | 558 | 7 | 0 | 134 | 0 | 0 |
| **ZnO.C-LC-84** | 119 | 385 | 0 | 0 | 87 | 0 | 0 |
| **ZnO.C-LC-105** | 145 | 558 | 0 | 0 | 79 | 0 | 36 |
| **ZnO.C-MC-21** | 218 | 667 | 0 | 0 | 113 | 0 | 0 |
| **ZnO.C-MC-42** | 122 | 407 | 0 | 0 | 106 | 0 | 0 |
| **ZnO.C-MC-63** | 156 | 527 | 0 | 0 | 96 | 0 | 0 |
| **ZnO.C-MC-84** | 150 | 475 | 0 | 0 | 100 | 0 | 11 |
| **ZnO.C-MC-105** | 45 | 209 | 0 | 0 | 16 | 0 | 0 |
| **ZnO.C-HC-21** | 227 | 596 | 0 | 82 | 128 | 0 | 0 |
| **ZnO.C-HC-42** | 104 | 212 | 0 | 77 | 38 | 0 | 0 |
| **ZnO.C-HC-63** | 92 | 544 | 0 | 72 | 36 | 0 | 0 |
| **ZnO.C-HC-84** | 109 | 328 | 0 | 38 | 34 | 0 | 0 |
| **ZnO.C-HC-105** | 44 | 265 | 0 | 51 | 26 | 0 | 0 |

**
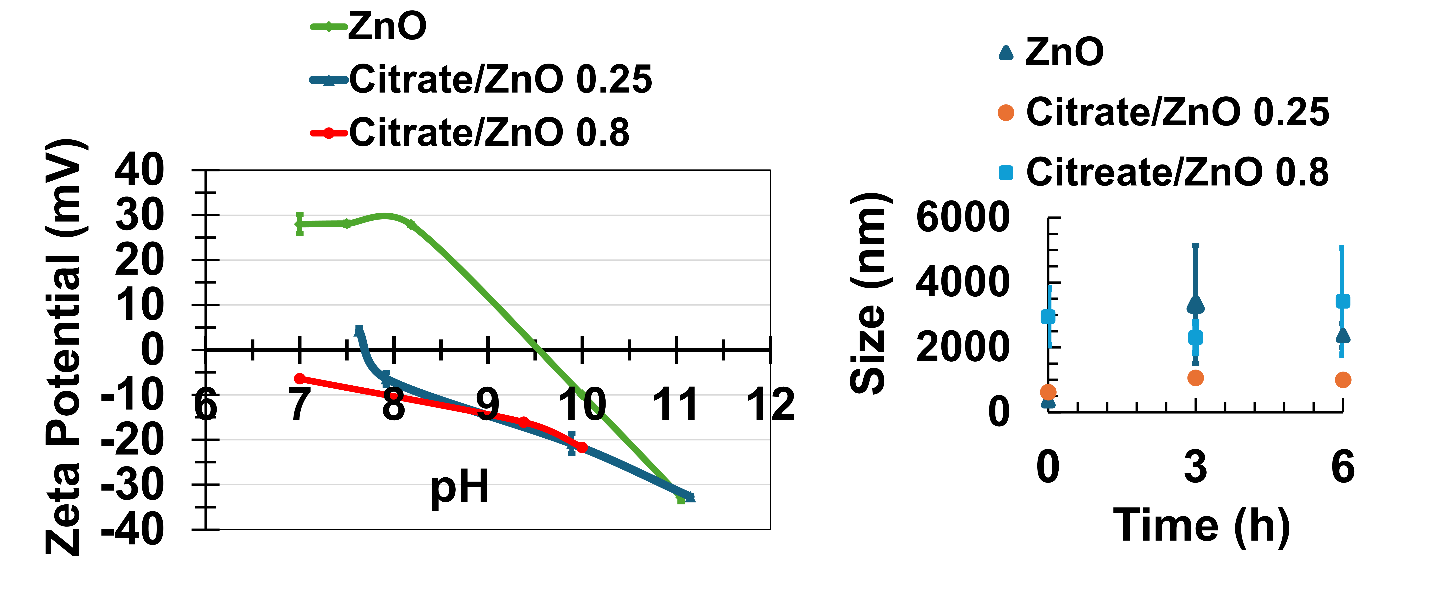

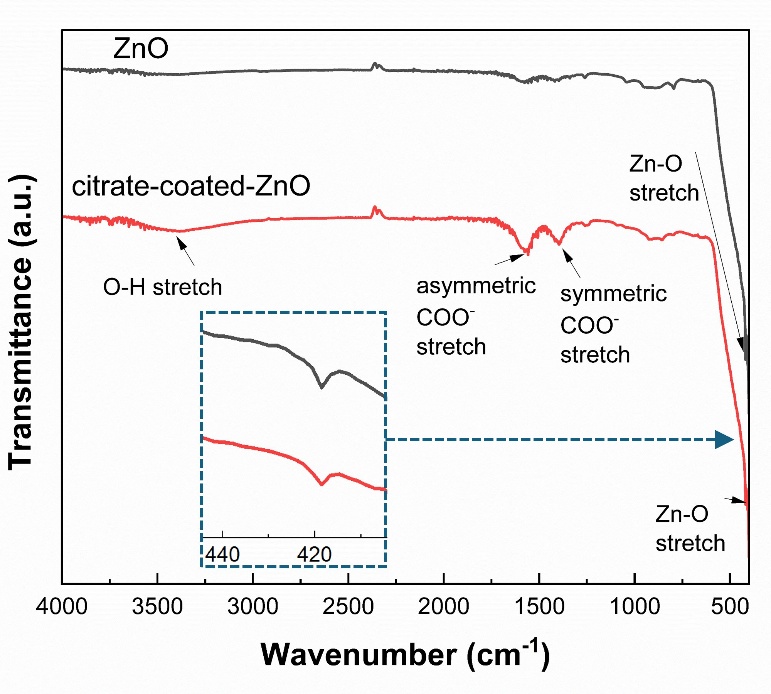
a)**

**b)**

**Figure S1.** (a) FTIR spectroscopy of pristine ZnO and citrate-coated ZnO nanoparticles (citrate-to-ZnO ratio of 0.25). The inset shows a zoomed-in view of the Zn–O stretching vibration peak. (b) Zeta potential and hydrodynamic diameter of ZnO and citrate-coated ZnO NPs.


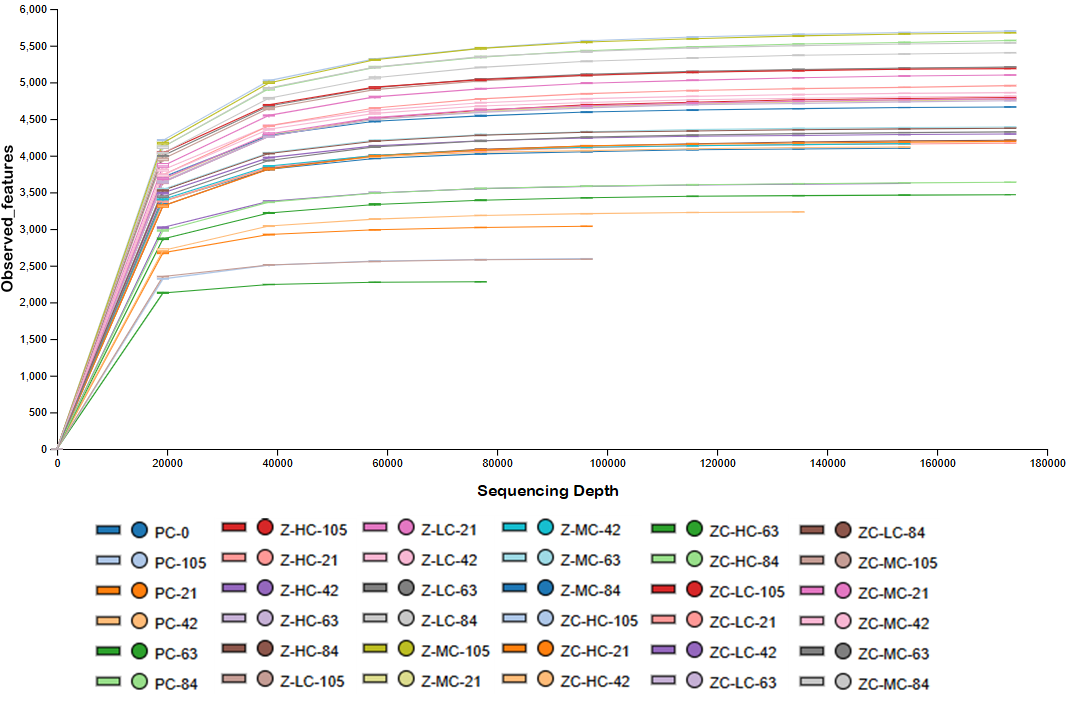


**Figure S2.** Rarefaction curves generated based on the normalized sequence reads (n=173,330) for the microbial communities of untreated and treated (ZnO and ZnO.C) soil samples.


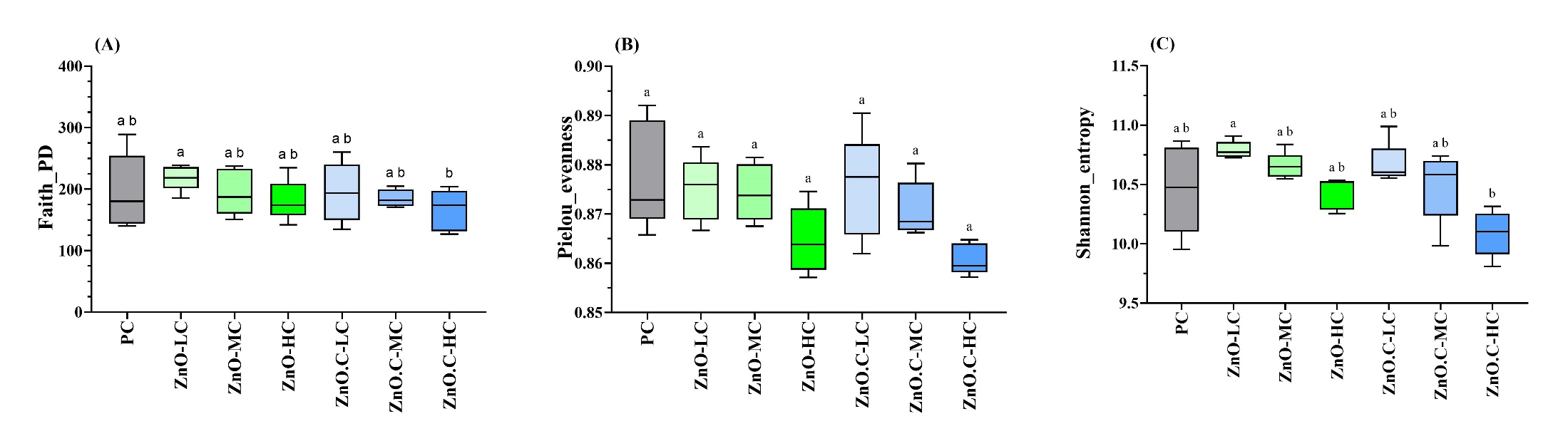


**Figure S3**. Alpha diversity matrices generated for the microbial communities of the untreated and treated (ZnO and citrate-ZnO NPs) soil samples. A) Faith’s Phylogenetic Diversity, (B) Pielou’s Evenness, and (C) Shannon Diversity Index. ‘PC” stands for positive control, ‘ZnO-LC’ for low concentration of ZnO NPs (0.01 mg/g of soil), ‘ZnO-MC’ for medium concentration of ZnO NPs (0.1 mg/g of soil), ‘ZnO-HC’ for high concentration of ZnO NPs (0.5 mg/g of soil), ‘ZnO.C-LC’ for low concentration of citrate-coated ZnO NPs (0.01 mg/g of soil), ‘ZnO.C-MC’ for medium concentration of citrate-coated ZnO NPs (0.1 mg/g of soil), ‘ZnO.C-HC’ for high concentration of citrate-coated ZnO NPs (0.5 mg/g of soil). Different letters (a, b) indicate a significant difference among soil treatments (Kruskal Wallis, p < 0.05).


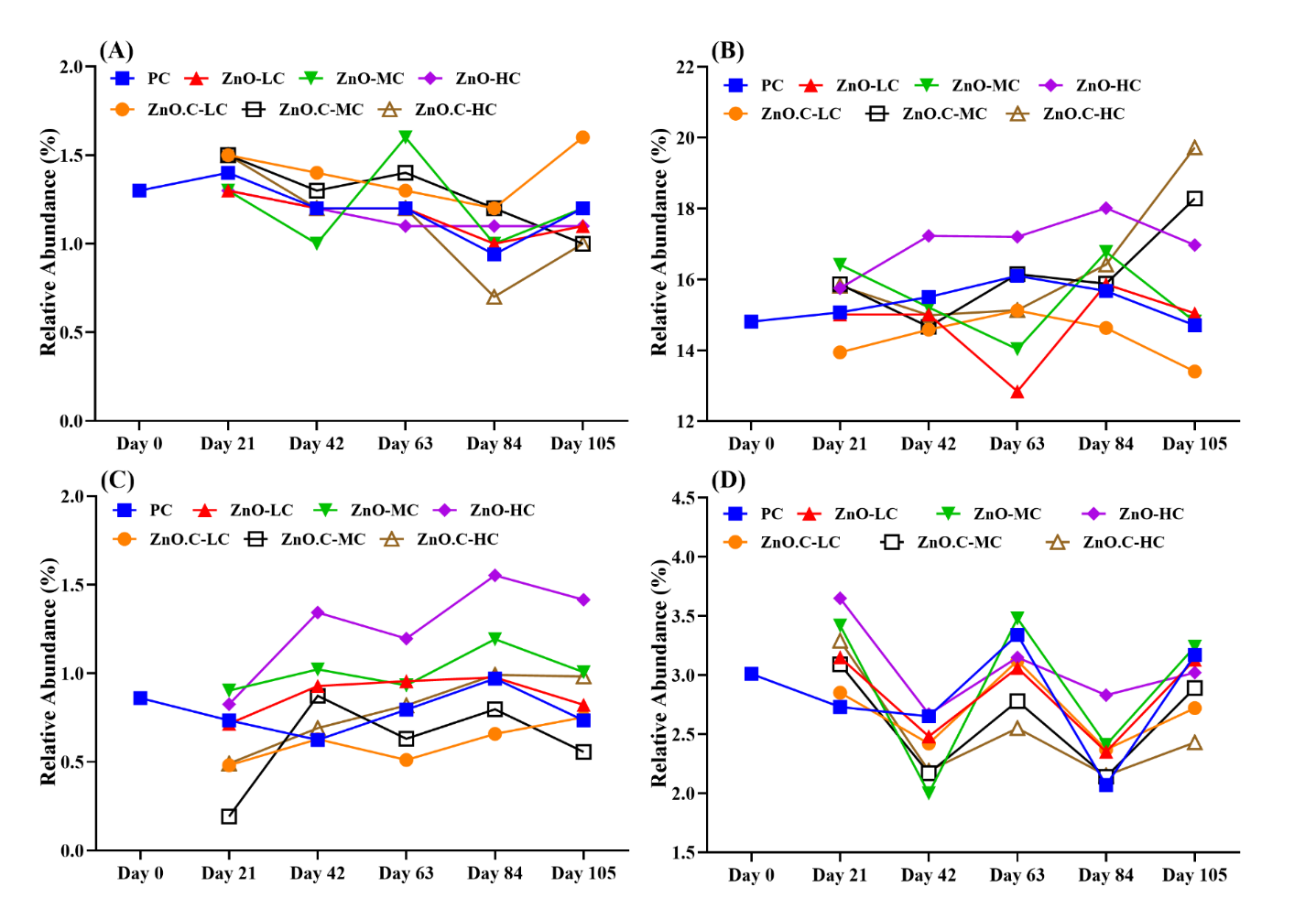


**Figure S4.** Relative abundance of nitrogen-metabolizing microorganisms in the untreated and treated (ZnO and citrate-ZnO) soil samples over time in various treatment groups. (A) Nitrogen-Fixing Microorganisms (NFM), (B) Ammonia-Oxidizing Microorganisms (AOM), (C) Nitrite-Oxidizing Microorganisms (NOM), and (D) Denitrifying Microorganisms (DM). ‘PC” stands for positive control, ‘ZnO-LC’ for low concentration of ZnO NPs (0.01 mg/g of soil), ‘ZnO-MC’ for medium concentration of ZnO NPs (0.1 mg/g of soil), ‘ZnO-HC’ for high concentration of ZnO NPs (0.5 mg/g of soil), ‘ZnO.C-LC’ for low concentration of citrate-coated ZnO NPs (0.01 mg/g of soil), ‘ZnO.C-MC’ for medium concentration of citrate-coated ZnO NPs (0.1 mg/g of soil), ‘ZnO.C-HC’ for high concentration of citrate-coated ZnO NPs (0.5 mg/g of soil).


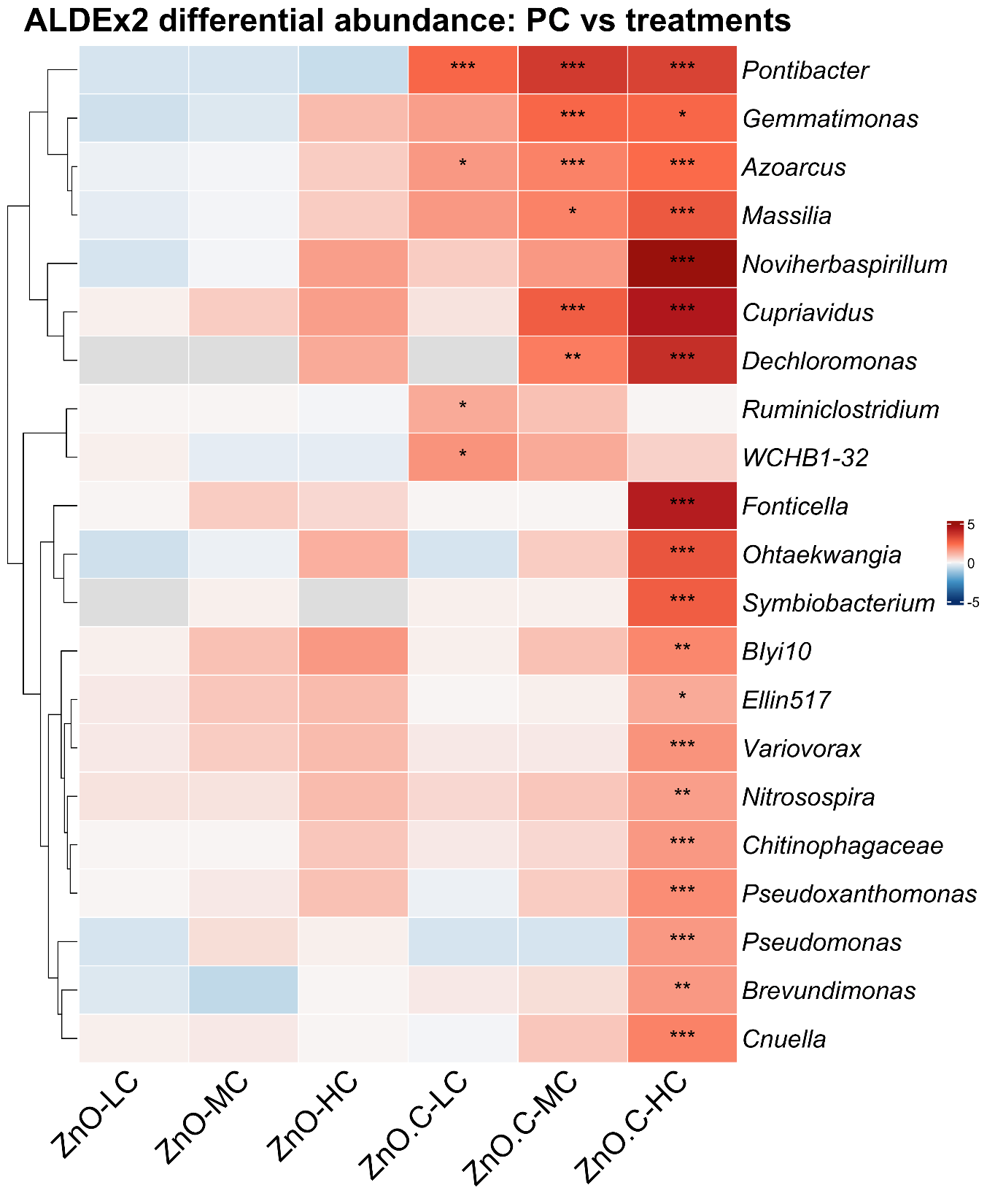


**Figure S5.** Differential abundance heatmap from ALDEx2 illustrating the top bacterial genera significantly influenced by ZnO and citrate-coated ZnO nanoparticle treatments compared to the positive control soil. Colors in the heatmap denote impact range, with red signifying enrichment and blue indicating depletion compared to the control. Asterisks stand for statistically significant differences determined by Benjamini–Hochberg adjusted p-values (p < 0.05, p < 0.01, p < 0.001). 'PC' is positive control, 'ZnO-LC' is low concentration ZnO (0.01 mg g⁻¹ soil), 'ZnO-MC' is medium concentration ZnO (0.1 mg g⁻¹ soil), 'ZnO-HC' is high concentration ZnO (0.5 mg g⁻¹ soil), while 'ZnO.C-LC', 'ZnO.C-MC', and 'ZnO.C-HC' denote low, medium, and high concentrations, respectively, of citrate-coated ZnO nanoparticles at the same concentrations.
